# ﻿Taxonomic study on Mysmenidae spiders (Mysmenidae, Araneae) from Xishuangbanna of Yunnan, China

**DOI:** 10.3897/zookeys.1124.85952

**Published:** 2022-10-10

**Authors:** Qiuqiu Zhang, Shuqiang Li, Yucheng Lin

**Affiliations:** 1 Key Laboratory of Bio-resources and Eco-environment (Ministry of Education), College of Life Sciences, Sichuan University, Chengdu, Sichuan 610064, China; 2 The Sichuan Key Laboratory for Conservation Biology of Endangered Wildlife, Sichuan University, Chengdu, Sichuan 610064, China; 3 Institute of Zoology, Chinese Academy of Sciences, Beijing 100101, China

**Keywords:** Diagnoses, discovery, minute clasping weavers, rainforest, types

## Abstract

Thirteen spider species belonging to the family Mysmenidae Petrunkevitch, 1928 are reported from Xishuangbanna Tropical Botanical Garden (XTBG), Menglun Township, Mengla County, Yunnan Province of China. One genus and five species are documented as new to science: *Mengmenabanna***gen. nov. et. sp. nov.** (♂♀), *Mengmenayulin***sp. nov.** (♀), *Mosuheguomu***sp. nov.** (♂♀), *Mysmenaluosuo***sp. nov.** (♂♀), and *Mysmenadai***sp. nov.** (♀). One species is proposed as a new combination: *Mosuzhengi* (Lin & Li, 2008) **comb. nov.** (♂♀, ex *Mysmena* Simon, 1894). The females of *Microdipoenamenglunensis* (Lin & Li, 2008), *Mysmenaarcilonga* Lin & Li, 2008, *Mysmenafurca* Lin & Li, 2008, and *Mysmenarostella* Lin & Li, 2008 are described for the first time. Three known species are re-examined and photographed: *Gaoligongataeniata* Lin & Li, 2014, *Mysmenabiangulata* (Lin & Li, 2008), and *Mysmenacornigera* (Lin & Li, 2008). Morphological diagnoses and illustrations are provided for these thirteen mysmenid species.

## ﻿Introduction

Xishuangbanna is a key biogeographic area and a biodiversity hotspot in China (Wang et al. 2020; Li et al. 2021a; Yao et al. 2021; Hong et al. 2022; Zhu et al. 2022). It shares a border with Myanmar in the southwest and Laos in the southeast and harbours more species diversity than typical tropical rain forests of Southeast Asia (Zhu et al. 2006). Implementing an “All Species Inventory” of spiders in Xishuangbanna Tropical Botanical Garden (XTBG, 1125-hectare area in total) has increased the spider species from fewer than 50 before 2006 to about 800 by the end of 2020 (Li 2020). The fifteen times increase in XTBG spider species during the past 15 years provides a striking example of high species richness within a small area.

Mysmenidae Petrunkevitch, 1928 is a small family of minute araneoids. Although widely distributed (except in the northern Holarctic realm, arid regions and Antarctica), Mysmenidae is still a poorly studied spider group in terms of faunal investigation and species diversity. These spiders live in cryptic habitats of moist leaf litter, mosses, and even dark caves in tropical and subtropical regions. Currently, 158 described species of 14 genera have been recorded worldwide (WSC 2022), of which nearly half of species have been discovered in the past two decades, including 38 species in eight genera from China. Lin and Li (2008) reported that 11 mysmenid species in China, of which eight came from XTBG. This is the only report of Mysmenidae spiders from this area so far, and most species were only described based on male specimens.

In the present paper, 13 species classified in five genera of mysmenid spiders from XTBG are recorded and illustrated, including five new species and one new genus. The goal of this paper is to provide detailed descriptions of these new taxa, to provide descriptions of the females of three known species for the first time, and to propose a new combination.

## ﻿Materials and methods

The inventory for this study included more than 800 spider specimens from XTBG belonging to the family Mysmenidae. Specimens were examined and measured in a 75% ethanol solution under a Leica M205 C stereomicroscope and photographed with a Canon EOS 60D wide zoom digital camera (8.5 megapixels) mounted on an Olympus BX 43 compound microscope. The digital photos were montaged using Helicon Focus 3.10 (Khmelik et al. 2006) image stacking software. Male palps and epigyna were examined and photographed after dissection. The left palp was photographed and described (if missing, the right was used). Epigyna were treated with lactic acid before being embedded in Hoyer’s gum and placed on an ultrathin slide to take photos of both sides of the vulva. All measurements are in millimetres. Leg measurements are given as follows: total length (femur, patella, tibia, metatarsus, and tarsus).

Abbreviations used in the text or figures are given in Table 1. References to figures in the cited papers are in lowercase (fig. or figs), figures in this paper are noted with an initial capital (Fig. or Figs). Apart from the type specimens of previously described species kept in **IZCAS**, all other examined morphological material is deposited in the **NHMSU** and **IZCAS**.

**Table 1.** List of abbreviations used in the figures or text.

Male palp		Vulva	
**AA**	apical apophysis on tegulum	**CD**	copulatory duct
**C**	conductor	**CO**	copulatory opening
**CT**	cymbial tooth	**EH**	epigynal hood
**Cy**	cymbium	**FD**	fertilization duct
**CyC**	cymbial conductor	**S**	spermathecae
**CyF**	cymbial fold	**Sp**	scape
**CyFs**	setae on cymbial fold	**Somatic morphology**	
**CyP**	cymbial process	**AER**	anterior eye row
**CyS**	cymbial serrula	**ALE**	anterior lateral eyes
**DK**	distal keel on cymbium	**AME**	anterior median eyes
**DL**	distal lobe on cymbium	**AP**	abdominal protuberance
**E**	embolus	**CS**	cheliceral spines on male
**MK**	median keel on cymbium	**FS**	femoral spot
**PC**	paracymbium	**MC**	metatarsal clasping spine
**SD**	spermatic duct	**PER**	posterior eye row
**Te**	tegulum	**PLE**	posterior lateral eyes
**Ti**	palp tibia	**PME**	posterior median eyes
		**TS**	tibial spine on male leg I
**Institutions**			
**IZCAS**	Institute of Zoology, Chinese Academy of Sciences, Beijing, China		
**NHMSU**	Natural History Museum of Sichuan University, Chengdu, China		
**XTBG**	Xishuangbanna Tropical Botanical Garden, Chinese Academy of Sciences, Mengla, China		

## ﻿Taxonomy

**Mysmenidae Petrunkevitch, 1928**

### *Gaoligonga* Miller, Griswold & Yin, 2009

#### ﻿ Gaoligonga taeniata

Taxon classificationAnimaliaAraneaeMysmenidae

Lin & Li, 2014

D8057AB8-D18B-5225-B02D-201C8BB5943E

Figs 1 , 2

Gaoligonga taeniata Lin and Li 2014: 178, figs 8A–F, 9A, B, 10A–D, 11A, B, 12A, B, 13A–D, 14A, B (♂♀).

##### Type material.

***Holotype*** ♂ (IZCAS) and ***paratypes*** 1♂ 5♀ (IZCAS), Vietnam: Ninh Binh, Cuc Phuong National Park, natural forest (20.410°N, 105.624°E; 436 m), by sieving leaf litter, 8.X.2007, D. Pham leg. Examined.

##### Other material examined.

4♂ 1♀ (MNHSU), China: Yunnan, Mengla, Menglun, XTBG, Rubber plantation (about 20 yr.) (22.038°N, 101.357°E; 586±9 m), by pitfall trapping, 16–31.V.2007, G. Zheng leg.; 2♂ (MNHSU) (22.090°N, 101.357°E; 585±10 m), China: Yunnan, Mengla, Menglun, XTBG, Rubber plantation (about 20 yr.), by pitfall trapping, 16–31.III.2007, G. Zheng leg.; 1♀ (MNHSU) (21.903°N, 101.282°E; 608±11 m), China: Yunnan, Mengla, Menglun, XTBG, the plantation of *Paramicheliabaillonii*, by pitfall trapping, 16–31.VI.2007, G. Zheng leg.

##### Diagnosis.

**Figure 1.**
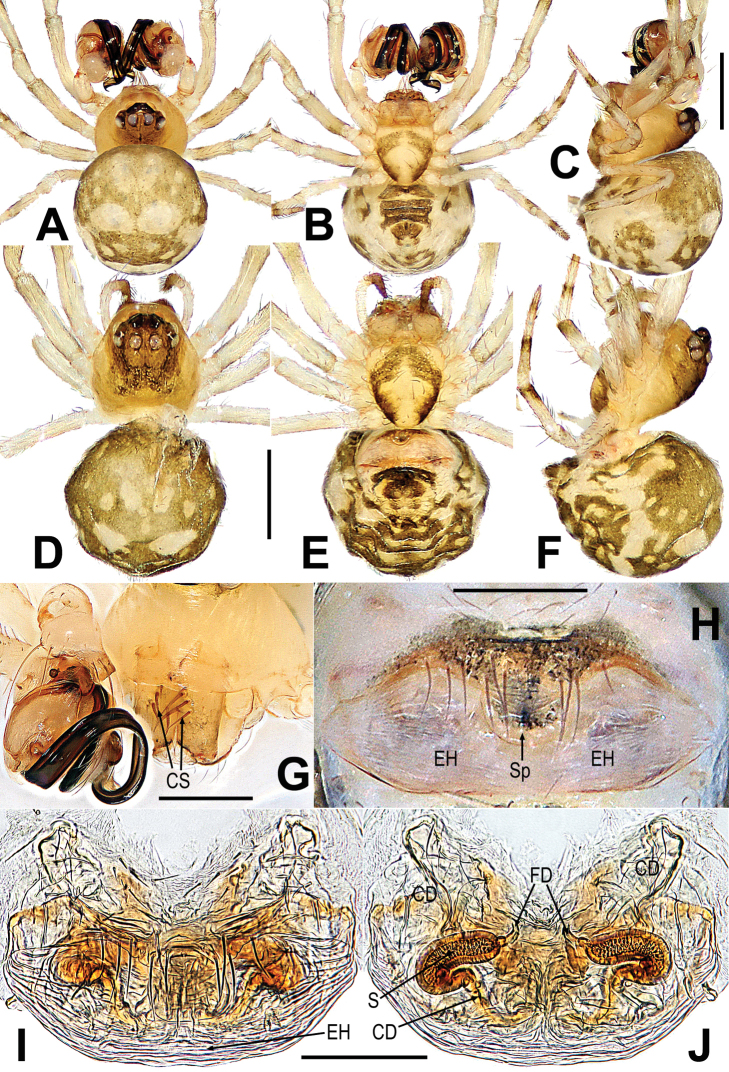
*Gaoligongataeniata***A–C** male habitus **D–F** female habitus **G** male prosoma **H** epigyne **I, J** vulva **A, D, J** dorsal **B, E, H, I** ventral **C, F** lateral **G** anterolateral. Abbreviations: CD = copulatory duct; CS = cheliceral spines on male; EH = epigynal hood; FD = fertilization duct; S = spermatheca; Sp = scape. Scale bars: 0.50 mm (**A–E**); 0.20 mm (**G**); 0.10 mm (**H–J**).

**Figure 2.**
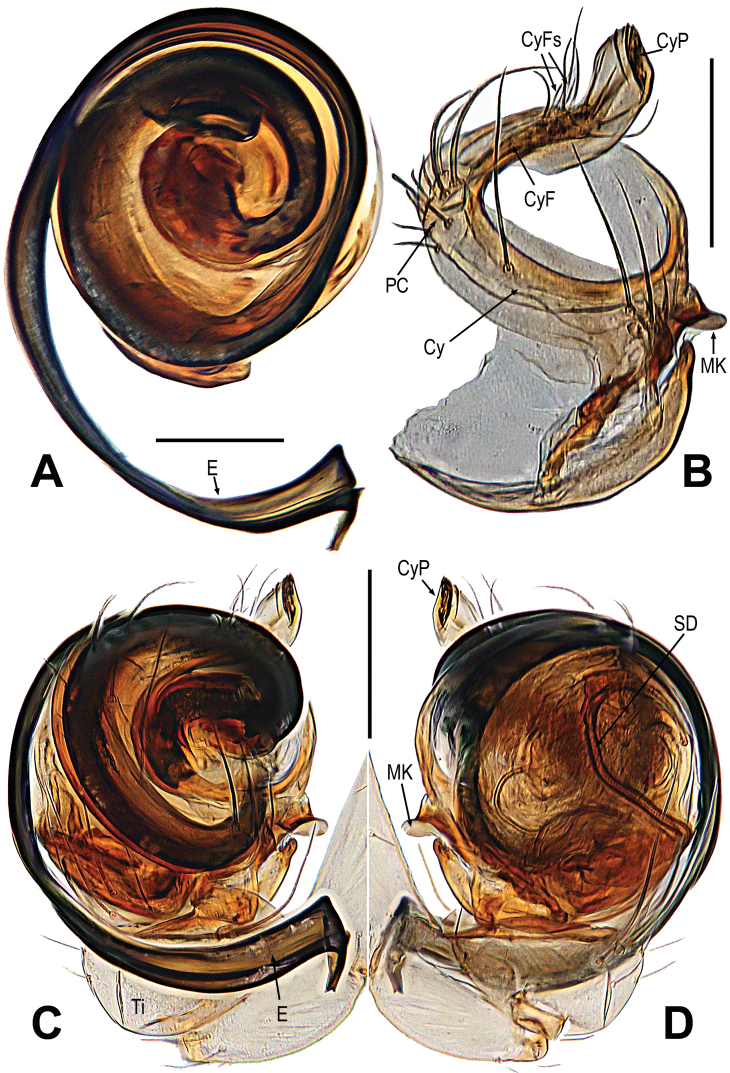
*Gaoligongataeniata***A** bulbus **B** cymbium **C, D** male palp. **A–C** prolateral **D** retrolateral. Abbreviations: Cy = cymbium; CyF = cymbial fold; CyFs = setae on cymbial fold; CyP = cymbial process; DL = distal lobe on cymbium; E = embolus; MK = median keel on cymbium; PC = paracymbium; SD = spermatic duct; Ti = palpal tibia. Scale bars: 0.10 mm (**A**); 0.20 mm (**B–D**).

This species can be distinguished from *G.changya* Miller, Griswold & Yin, 2009 (Miller et al. 2009: 48, figs 38A–E, 39A, B, 40A–F, 41A, B, 43A, B) and *G.zhusun* Miller, Griswold & Yin, 2009 (Miller et al. 2009: 50, figs 43D, E, 44A–E, 45A, B, 46A–D, 47D) by the male having three frontal spines near the base of each chelicera (Fig. 1G vs. fig. 38A, 40E, 44A, 46E, Miller et al. 2009: 119, 421, 125, 127), the palp having a “S”-shaped cymbium and median keel on cymbium, lacking basal keel and tooth on cymbium (Fig. 2B, D vs. fig. 39A–B, 40A–C, 45A–B, 46A–C, Miller et al. 2009: 120, 121, 126, 127), and the strong, anticlockwise spiral embolus with a tortuous end (Fig. 2A, C vs. fig. 39A–B, 45A–B, Miller et al. 2009: 120, 126). Females can be distinguished by the large, long central knob-shaped scape (Fig. 1H vs. fig. 43B, E, Miller et al. 2009: 124), having a saccular epigynal hood, the nearly transversely clubbed spermathecae, and the membranous, broad and complicated copulatory ducts (Fig. 1J vs. fig. 43B, E, Miller et al. 2009: 124).

##### Description.

See Lin and Li (2014: 178), and Figs 1, 2.

##### Distribution.

China (Yunnan), Vietnam.

##### Remark.

This species is newly recorded in XTBG, China.

#### ﻿ Mengmena

Taxon classificationAnimaliaAraneaeMysmenidae

Lin & Li gen. nov.

59EB5BC5-4A89-5439-BA8A-90651B53A446

https://zoobank.org/91BD56B2-545A-4D61-86DF-19D0ED9671BF

##### Type species.

*Mengmenabanna* Lin & Li, sp. nov.

##### Etymology.

The generic name is a combination of the first four letters of Menglun (type locality of type species) and the latter half of *Mysmena*. The gender is feminine.

##### Diagnosis.

**Figure 3.**
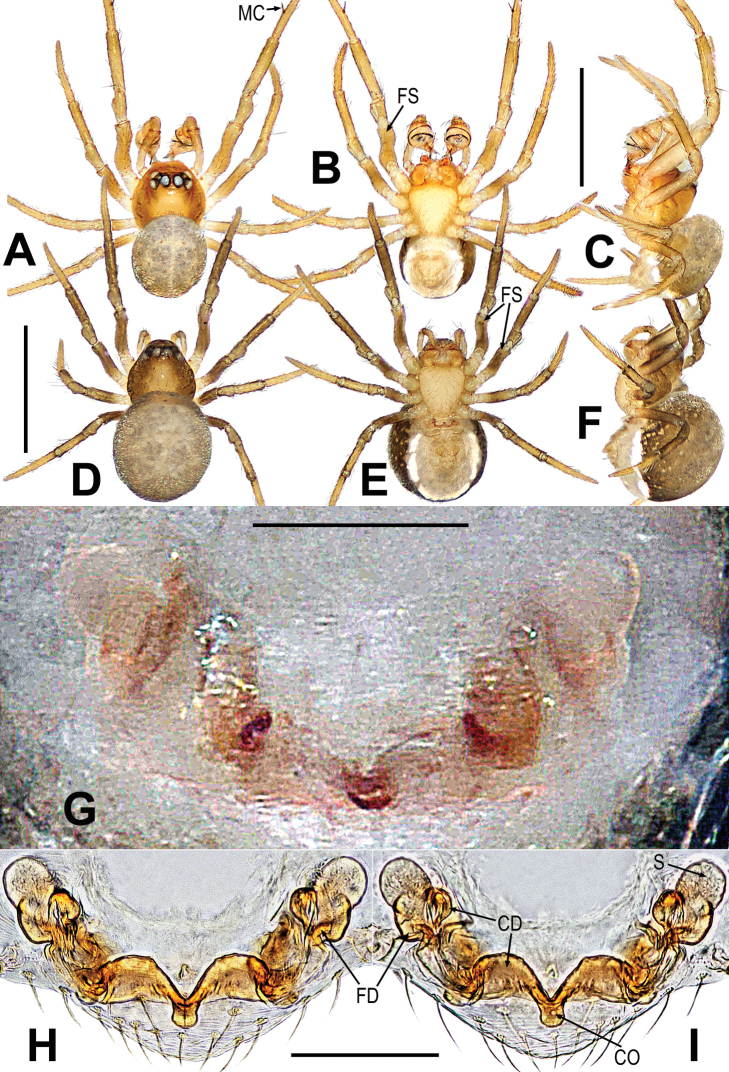
*Mengmenabanna* sp. nov. **A–C** male habitus **D–F** female habitus **G** epigyne **H, I** vulva **A, D, I** dorsal **B, E, G, H** ventral **C, F** lateral. Abbreviations: CD = copulatory duct; CO = copulatory opening; FD = fertilization duct; FS = femoral spot; MC = Metatarsal clasping spine; S = spermatheca. Scale bars: 0.50 mm (**A–F**); 0.10 mm (**G–I**).

**Figure 4.**
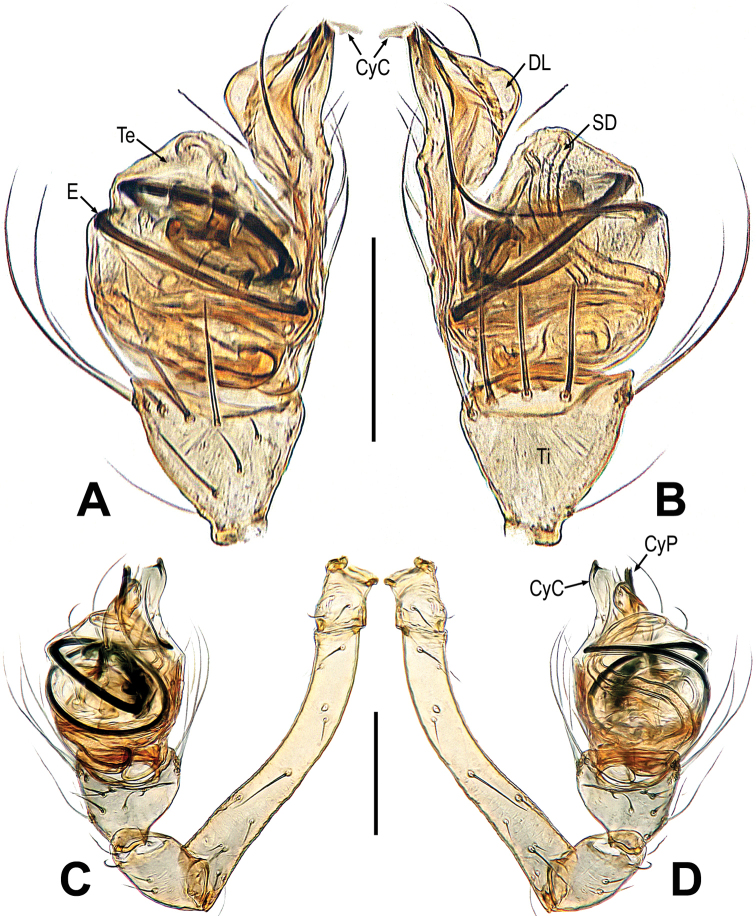
*Mengmenabanna* sp. nov. **A–D** male pale. **A** dorsal **B** ventral **C** prolateral **D** retrolateral. Abbreviations: CyC = cymbial conductor; CyP = cymbial process; DL = distal lobe on cymbium; E = embolus; SD = spermatic duct; Te = tegulum; Ti = palpal tibia. Scale bars: 0.20 mm.

**Figure 5.**
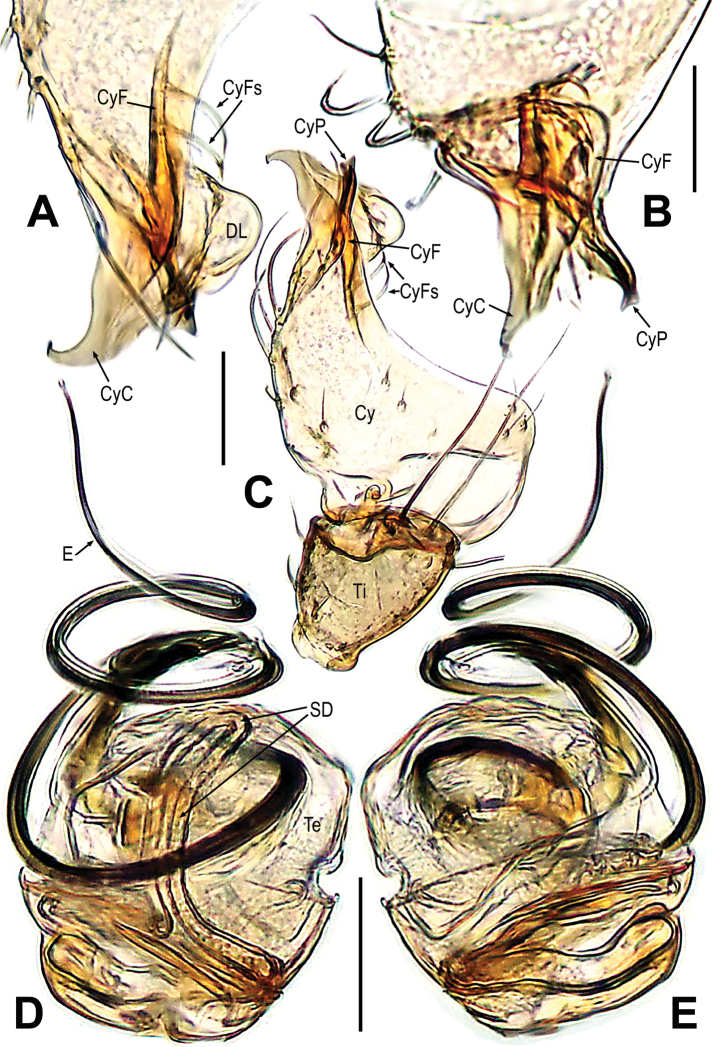
*Mengmenabanna* sp. nov. **A, B** cymbial terminals **C** palpal cymbium and tibia **D, E** bulbus. **A** prodorsal **B** retroventral **C** retrodorsal **D** ventral **E** dorsal. Abbreviations: Cy = cymbium; CyC = cymbial conductor; CyF = cymbial fold; CyFs = setae on cymbial fold; CyP = cymbial process; DL = distal lobe on cymbium; E = embolus; SD = spermatic duct; Te = tegulum; Ti = palpal tibia. Scale bars: 0.05 mm (**A, B**); 0.20 mm (**C**); 0.10 mm (**D, E**).

**Figure 6.**
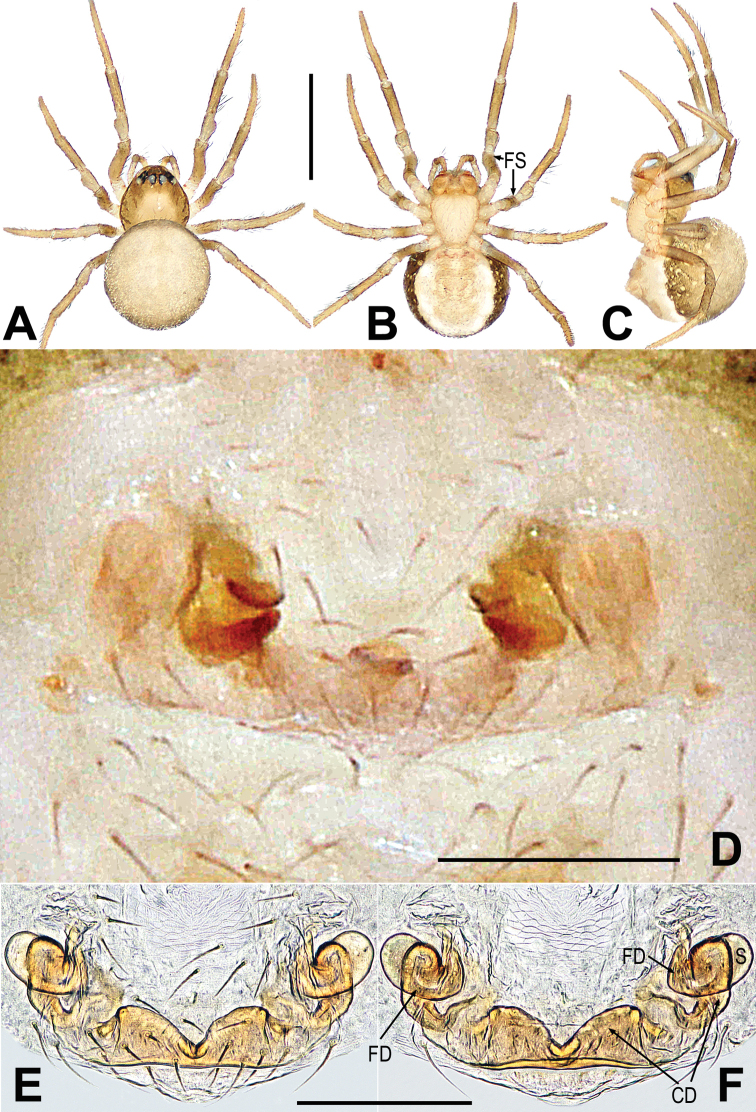
*Mengmenayulin* sp. nov. **A–C** female habitus **D** epigyne **E, F** vulva. **A, F** dorsal **B, D, E** ventral **C** lateral. Abbreviations: CD = copulatory duct; FD = fertilization duct; FS = femoral spot; S = spermatheca. Scale bars: 0.50 mm (**A–C**); 0.10 mm (**D–F**).

The *Mengmena* gen. nov. can be easy distinguished from other mysmenids, except *Mysmeniola* Thaler, 1995, by lacking anterior median eyes in both sexes (Figs 3A, D, 6A). It resembles *Mysmeniola* in having six eyes (anterior median eyes absent), a submesial mating clasper on metatarsus I of males, and a long filiform embolus extending to the distal tip of the cymbium, but differs from *Mysmeniola* in lacking a cluster of strong spines at the base of the male clypeus (*Mysmeniola*, Thaler 1995: figs 1, 2), and lacking a prolateral apical process on male palpal tibia (Thaler 1995: fig. 5). In addition, the male can be distinguished from other mysmenids by the complex structure of the apical part of cymbium (Figs 5A–C). The cymbium tip specialized as a triangular cymbial conductor (Figs 4A–B, 5A–C), and the retrolateral base of cymbial conductor present a distal lobe (Figs 4B, 5A), the cymbial fold originated from the base of cymbial conductor and the cymbial fold distal end extended anteriorly form a scleroticed cymbial process (Figs 4D, 5A–C); the absence of cymbial spur (or cymbial tooth) and paracymbium (Figs 4A–D, 5C). The female can be distinguished by the widely separated spermathecae (at least 4× their width, 2–3× in other mysmenids), and copulatory opening situated at the union of copulatory ducts (Figs 3H, I, 6E, F).

##### Description.

Body bicolour, dorsally grey, ventrally yellow or pale yellow (Figs 3A–F, 6A, B). Anterior median eyes absent (Figs 3A, 3D, 6A). Abdomen without posterior tubercle (Figs 3A–D, 6A–C). Male cephalic area moderately elevated, tibia I without prolateral macrosetae (Fig. 3C). Femoral spots present on leg I of the males and legs I–II of the females (Figs 3B, 3E, 6B).

***Male palp***: cymbium oriented ventrally on the palp (Fig. 4A, B). Cymbial spur and paracymbium absent (Figs 4A–D, 5C). Cymbial process arising from the cymbial fold at apex, strongly sclerotized (Figs 4D, 5A–C). Cymbial conductor wide (Fig. 5A–C). Cymbial fold long and sclerotized, from the base of cymbial conductor (Fig. 5C). Distal lobe on retrolateral tip of cymbium (Fig. 4A, B). Embolus threadlike, coiled with at least two loops (Figs 4A–D, 5D–E).

***Epigyne***: weakly sclerotized (Figs 3G, 6D). Scape absent (Figs 3G–I, 6D–F). Spermathecae ovate or slightly twisted, separated by at least four times their width. Copulatory ducts wide, shape convoluted. Copulatory opening small hole-shaped or arc shape, situated at the union of copulatory ducts (Figs 3H, I, 6E, F).

##### Composition.

*Mengmenabanna* sp. nov. and *M.yulin* sp. nov.

##### Distribution.

China (Yunnan).

#### ﻿ Mengmena banna

Taxon classificationAnimaliaAraneaeMysmenidae

Lin & Li sp. nov.

66A03AC2-B26B-58A4-BB35-8D6C5B48F392

https://zoobank.org/66FB56DD-4BF3-487E-8412-9F95DF392C58

Figs 3 , 4 , 5

##### Type material.

***Holotype*** ♂ (IZCAS) and ***paratypes*** 3♂ 4♀ (IZCAS), China: Yunnan: Mengla County, Menglun Town, Xishuangbanna National Nature Reserve, the primary tropical seasonal rain forest (21.957°N, 101.217°E; 744±15 m), 16–31.I.2007, by pitfall trapping, G. Zheng leg.; 6♂ 25♀ (NHMSU), China: Yunnan: Mengla County, Menglun Town, XTBG, in the plantation of *Paramicheliabaillonii* (about 20 yr.) (21.903°N, 101.282°E; 608±11 m), 5–12.IX.2006, by searching, G. Zheng leg.

##### Other material examined:

11♂ 33♀ (IZCAS), China: Yunnan, Mengla County, Menglun Town, Xishuangbanna National Nature Reserve, the plantation of *Paramicheliabaillonii* (21.956°N, 101.523°E; 608±11 m), 5–12.XI.2006, by search collecting, G. Zheng leg.

##### Etymology.

The specific name derives from the type locality; noun in apposition.

##### Diagnosis.

*Mengmenabanna* sp. nov. can be distinguished from its congener *M.yulin* sp. nov. by both sides of the copulatory duct being fused at the midline position and forming a V-shaped structure, and the copulatory opening situated below the bottom of the V-shaped structure (Fig. 3H, I vs. Fig. 6E, F).

##### Description.

**Male. *Measurements***: total length 0.58. Prosoma 0.30 long, 0.28 wide, 0.29 high. Abdomen 0.34 long, 0.31 wide, 0.38 high. Clypeus 0.07 high. Sternum 0.20 long, 0.19 wide. Length of legs: I 0.91 (0.31, 0.12, 0.21, 0.11, 0.16); II 0.80 (0.25, 0.11, 0.18, 0.10, 0.16); III 0.62 (0.18, 0.09, 0.11, 0.11, 0.13); IV 0.72 (0.22, 0.10, 0.15, 0.12, 0.13).

***Somatic characters*** (Fig. 3A–C). ***Coloration***: prosoma light-yellow centrally, deep yellow marginally. Ocular base black. Chelicera yellow, endites and labium yellow, the sternum light-yellow. Abdomen silver grey dorsally, yellow with a “U”-shaped white and a “U”-shaped brown stripe ventrally. Legs yellow. ***Prosoma***: carapace near round. Cephalic part slightly elevated. Ocular area at apex. AME absent, six eyes in two rows. ALE and PLE contiguous. PER slightly recurved. Sternum scutiform, plump, covered with sparse setae. ***Legs***: covered with setae and bristles. The leg I with a mating clasper on distal 1/3 position of metatarsus and a subdistal sclerotized femoral spot present at surface of ventral femur. ***Abdomen***: near round in dorsum.

***Palp*** (Figs 4–5): weakly sclerotized. The tibia cup-shaped, covered with long setae along distal brim (Fig. 4A, B). Cymbium membranous, with a distal lobe on cymbium (Figs 4A, B, 5A–C). Cymbial conductor triangular, distal end hook-shaped (Figs 4A–D, 5A–C). Cymbial process straight but the tip recurved, situated on dorsal cymbial conductor (Figs 4D, 5A–C). Cymbial fold sclerotized, derived from the base of cymbial process, and bears a row of ordered setae (Fig. 5A, C). The absence of paracymbium (Fig. 5C). Tegulum smooth, translucent; spermatic duct visible from the tegulum (Figs 4B, D, 5D). Embolus threadlike, coiled into two crossed loops (Figs 4A–D, 5D, E).

**Female. *Measurements***: total length 0.64. Prosoma 0.29 long, 0.27 wide, 0.27 high. Abdomen 0.42 long, 0.40 wide, 0.46 high. Clypeus 0.06 high. Sternum 0.20 long, 0.19 wide. Length of legs: I 0.87 (0.27, 0.12, 0.18, 0.14, 0.16); II 0.75 (0.22, 0.11, 0.15, 0.12, 0.15); III 0.61 (0.17, 0.09, 0.11, 0.11, 0.13); IV 0.72 (0.21, 0.10, 0.14, 0.13, 0.14).

***Somatic characters*** (Fig. 3D–F). ***Coloration***: prosoma light-brown centrally, deep brown marginally. Ocular base black. Chelicera, endites, labium and sternum light-brown. Abdomen silver grey, with a “U”-shaped white stripe. Legs brown. ***Prosoma***: carapace pear-shaped. Ocular pattern as in male. AME absent, six eyes in two rows, the PER slightly recurved, the ALE and PLE contiguous. Sternum scutiform, plump, covered with sparse setae. ***Legs***: covered with setae and bristles, a sclerotized femoral spot present at apical ventral surface of leg I and II. ***Abdomen***: same as in male.

***Epigyne*** (Fig. 3G–I): weakly sclerotized, covered with sparse short setae along ventral brim (Fig. 3G, H). Internal structures indistinctly visible from translucent cuticle (Fig. 3G). Spermathecae small, ovate, widely separated by at least four times their width (Fig. 3H, I). Fertilization ducts short, winding, arising from ventral of the spermathecae (Fig. 3H, I). Copulatory ducts wide, both sides of copulatory duct fused at the midline position and formed a V-shaped structure; the copulatory opening situated below the bottom of the V-shaped structure (Fig. 3H, I).

##### Distribution.

Known only from the type locality.

#### ﻿ Mengmena yulin

Taxon classificationAnimaliaAraneaeMysmenidae

Lin & Li sp. nov.

43A6E235-4202-5D22-B586-0FDB0AC8CA4D

https://zoobank.org/5EE0208C-62D5-46A6-83FE-F9FD50A9A5EF

Fig. 6

##### Material examined.

***Holotype*** ♀ (IZCAS) and ***paratypes*** 4♀ (IZCAS), China: Yunnan, Mengla County, Menglun Town, XTBG, the secondary tropical rainforest (22.036°N, 101.389°E; 598±17 m), 9–13.VIII.2006, by pitfall trapping, G. Zheng leg.; 31♀ (NHMSU), same data as holotype, 1–15.VII.2007, by searching, G. Zheng leg.

##### Etymology.

The specific name derives from the Chinese pinyin for rainforest (yǔ lín), refers to it living in rainforest habitats. The epithet is a noun in apposition.

##### Diagnosis.

The new species is similar to *Mengmenabanna* sp. nov. but can be distinguished by having a straight and smooth posterior brim formed by the fusion of the copulatory ducts, and the copulatory opening situated above this brim (Fig. 6E, F vs. Fig. 3H, I).

##### Description.

**Female. *Measurement***: total length 0.78. Prosoma 0.32 long, 0.29 wide, 0.28 high. Abdomen 0.49 long, 0.48 wide, 0.53 high. Clypeus 0.05 high. Sternum 0.21 long, 0.20 wide. Length of legs [total length (femur, patella, tibia, metatarsus, tarsus)]: I 0.92 (0.30, 0.11 0.19, 0.15, 0.17); II 0.85 (0.28, 0.10, 0.17, 0.13, 0.17); III 0.66 (0.19, 0.09, 0.12, 0.11, 0.15); IV 0.79 (0.25, 0.10, 0.16, 0.12, 0.16).

***Somatic characters*** (Fig. 6A–C). ***Coloration***: prosoma light-yellow centrally, deep yellow marginally. Ocular base black. Chelicera yellow. Endites, labium and sternum light-yellow. Abdomen grey dorsally, pale yellow ventrally, brown marginally. Legs yellow. ***Prosoma***: carapace pear-shaped. Cephalic part slightly elevated. AME absent, six eyes in two rows, white, with black rings. ALE and PLE contiguous. Sternum scutiform, plump, covered with sparse setae. ***Legs***: covered with setae and bristles, a sclerotized femoral spot present at apical ventral surface of leg I and II. ***Abdomen***: round in dorsum.

***Epigyne*** (Fig. 6D–F): spermathecae small, ovate, widely separated by at least four times their width (Figs 6E, F). Fertilization ducts short, derived from inner side of the spermathecae and bent anteriorly to form an arc (Fig. 6F). Copulatory ducts arising from ventral of spermathecae, both sides of copulatory duct fused at the midline position and forming two symmetrical peak shapes (the anterior brim of the fused copulatory ducts broad arc shape, the posterior brim of the fused copulatory ducts near straight) (Fig. 6E, F). Copulatory opening inconspicuous, situated above the straight brim (Fig. 6E, F).

**Male.** Unknown.

##### Distribution.

Known only from the type locality.

### *Microdipoena* Banks, 1895

#### ﻿ Microdipoena menglunensis

Taxon classificationAnimaliaAraneaeMysmenidae

(Lin & Li, 2008)

09CB9238-476C-5449-B6AE-D1A8464A8963

Figs 7 , 8 , 9

Mysmenella menglunensis Lin and Li 2008: 506, fig. 13A–I (♂).
Microdipoena menglunensis Lopardo and Hormiga 2015: 783.

##### Type material.

***Holotype*** ♂ (IZCAS), China: Yunnan, Mengla, XTBG (21.913°N, 101.267°E; 556±11 m), by pitfall trapping, 18.VII.2007, Guo Zheng leg.; ***paratypes*** 1♂ (IZCAS), same site as for preceding, Rubber plantation (21.908°N, 101.266°E; 569±11 m), 21.VII.2007; 1♂, same site as for preceding, Primary tropical seasonal rainforest (21.917°N, 101.275°E; 558±17 m), 22.VII.2007, G. Zheng. Examined.

##### Other material examined.

3♂ 5♀ (MNHSU), China: Yunnan, Mengla, Menglun, XTBG, Rubber-Tea plantation (about 20 yr.) (22.029°N, 101.522°E; 569±11 m), by pitfall trapping, 16–31.V.2007, G. Zheng leg.; 3♂ 3♀ (MNHSU), China: Yunnan, Mengla, Menglun, XTBG, Rubber plantation (about 20 yr.) (22.038°N, 101.357°E; 586±9 m), by searching, 4–11.IV.2007, G. Zheng leg.

##### Diagnosis.

This species is similar to *Microdipoenajobi* (Kraus, 1967) and *Microdipoenasamoensis* (Marples, 1955), but can be distinguished by the detailed structures of the embolus; this species has a distal lobe on the cymbium apex and has a sclerotized cymbial fold bore a row of ordered setae (Fig. 8A, C–F vs. fig. 132D–F, Lopardo and Hormiga 2015: 675). The female distinguished by the semicircular spermathecae separated by 2.5 times their diameter, near globular in *M.jobi* and *M.samoensis* (Fig. 9C, D vs. fig. 129E, F, Lopardo and Hormiga 2015: 672)

##### Description.

**Figure 7.**
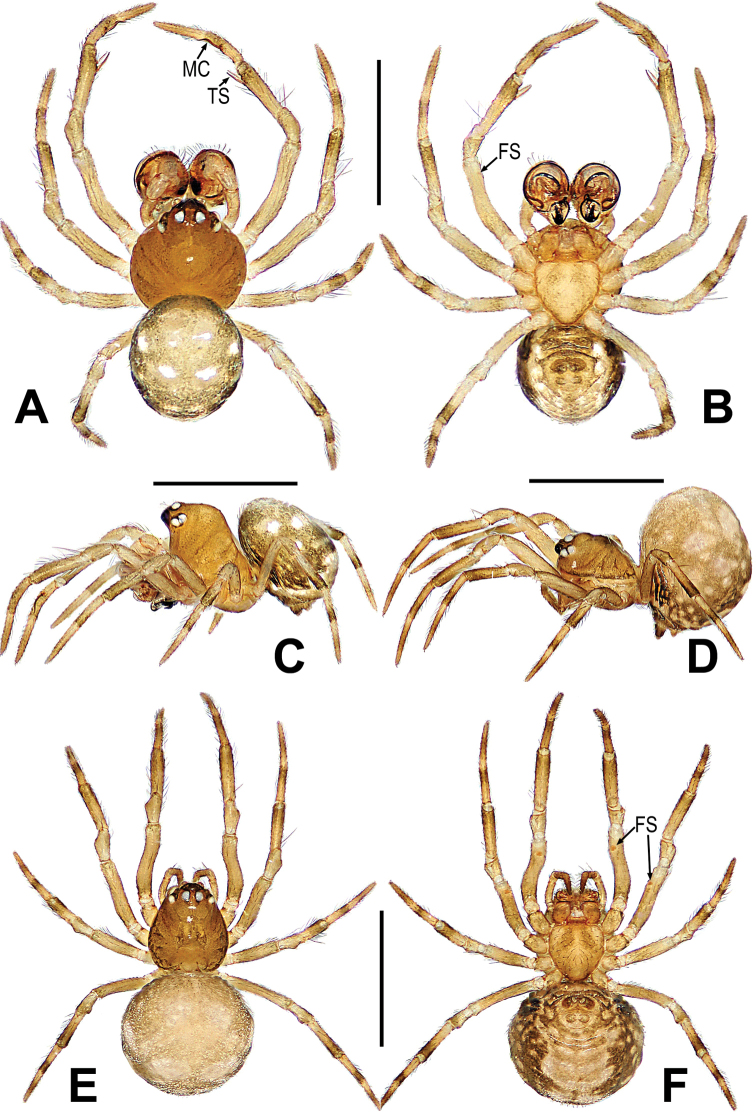
*Microdiponeamenglunensis***A–C** male habitus **D–F** female habitus. **A, E** dorsal **B, F** ventral **C, D** lateral. Abbreviations: FS = femoral spot; MC = Metatarsal clasping spine; TS = tibial spine on male leg I. Scale bars: 0.50 mm.

**Male.** See Fig. 7A–C and Lin and Li (2008): 506.

**Figure 8.**
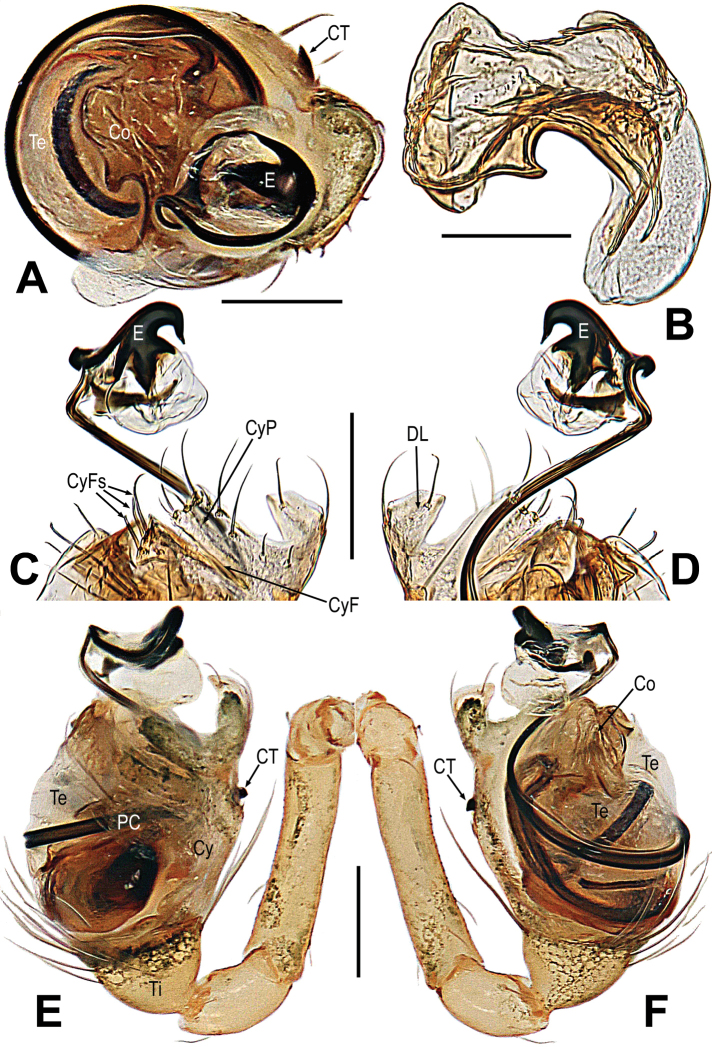
*Microdiponeamenglunensis***A, E, F** male palp **B** conductor **C, D** embolus and cymbial terminal **A** apical **B** dorsal **C, E** prolateral **D, F** retrolateral. Abbreviations: C = conductor; CT = cymbial tooth; Cy = cymbium; CyF = cymbial fold; CyFs = setae on cymbial fold; CyP = cymbial process; DL = distal lobe on cymbium; E = embolus; PC = paracymbium; SD = spermatic duct; Te = tegulum; Ti = palpal tibia. Scale bars: 0.10 mm (**A, C–F**); 0.05 mm (**B**).

***Palp*** (Fig. 8A–F): orange; tibia small, cup-shaped, except for retrolateral region, a row of long setae almost encircling the distal brim (Fig. 8E, F). Cymbium nearly transparent, with a large cymbial tooth at the ventral median, a distal lobe and a cymbium process on the cymbium apex; the cymbial fold slightly sclerotized, bore a row of ordered setae (Fig. 8A, C–F). Paracymbium wider, tongue-shaped, with long setae (Fig. 8E). The bulb is embedded in a translucent membranous tegulum. Embolus very long, coiled into two crossed loops; the apical structure of the embolus considerably complicated (Fig. 8C–F).

##### New morphological data.

**Female. *Measurements***: total length 0.81. Prosoma 0.24 long, 0.20 wide, 0.20 high. Abdomen 0.57 long, 0.57 wide, 0.62 high. Clypeus 0.06 high. Sternum 0.18 long, 0.16 wide. Length of legs: I 0.92 (0.30, 0.12, 0.28, 0.12, 0.10); II 0.90 (0.28, 0.12, 0.26, 0.14, 0.10); III 0.70 (0.26, 0.08, 0.16, 0.10, 0.10); IV 0.94 (0.32, 0.12, 0.28, 0.12, 0.10).

***Somatic characters*** (Fig. 7D–F). ***Coloration***: same as in male. ***Prosoma***: carapace long, nearly pear-shape. Cephalic part lower than in male. Ocular pattern as in male. Chelicerae, endites, labium and sternum as in male. ***Legs***: covered with setae and bristles, a sclerotized subdistal-ventral femoral spot present on surface of legs I and II. ***Abdomen***: same as in male.

**Figure 9.**
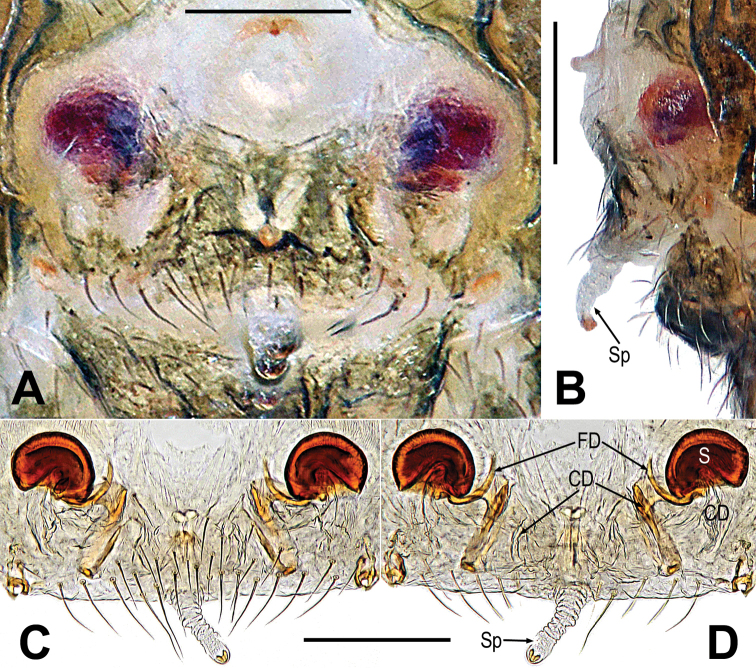
*Microdiponeamenglunensis***A, B** epigyne **C, D** vulva. **A, C** ventral **B** lateral **D** dorsal. Abbreviations: CD = copulatory duct; FD = fertilization duct; S = spermatheca; Sp = scape. Scale bars: 0.10 mm.

***Epigyne*** (Fig. 9A–D): the structure can be seen through the cuticle (Fig. 9A–D). Scape long, curved, with narrow folds (Fig. 9B–D). Spermathecae large, semicircular, separated by 2.5 times their diameter (Fig. 9C, D). Fertilization ducts short, bending anteriorly, arising from lower edge of spermathecae (Fig. 9C, D). Copulatory ducts membranous, slightly sclerotized, coiled posterior of spermathecae, connected above the spermathecae (Fig. 9C, D).

##### Distribution.

Southwestern China (Yunnan).

##### Remarks.

The female of *M.menglunensis* is described for the first time.

### *Mosu* Miller, Griswold & Yin, 2009

#### ﻿ Mosu heguomu

Taxon classificationAnimaliaAraneaeMysmenidae

Lin & Li sp. nov.

4187AB2A-D68E-5D96-ACD3-954929A2DBF5

https://zoobank.org/8A572298-31C4-4011-B966-B8D26E9283CA

Figs 10 , 11 , 12

##### Type material.

***Holotype*** ♂ (IZCAS) and ***paratypes*** 2♂ 4♀ (IZCAS), China: Yunnan, Mengla, Menglun, XTBG, *Paramicheliabaillonii* plantation (about 20 yr.) (22.114°N, 101.279°E; 556±11 m), by pitfall trapping, 1–15.II.2007, G. Zheng leg. Examined.

##### Other material examined.

4♂ 12♀ (NHMSU), China: Yunnan, Jinghong, Mengla, Mengyuan, Chengzi village, Buffalo Cave Scenic Spot, entrance of 3^#^ cave (21.798°N, 101.399°E; 716 m), by searching, 16.VIII.2011, G. Zheng and Y. Lin leg.

##### Etymology.

The specific name derives from a Chinese Pinyin “hé guŏ mù”, referring to the Chinese name of *Paramicheliabaillonii*.

##### Diagnosis.

Male differed from other congeners by the male palp with a cymbial serrula, a distal lobe and median keel on the cymbium, and cymbial tooth on the median cymbium (Fig. 11C). Female can be distinguished by the sclerotized and expanded copulatory opening at the tip of scape (Fig. 11C, D).

##### Description.

**Male. *Measurements***: total length 1.06. Prosoma 0.34 long, 0.5 wide, 0.5 high. Abdomen 0.7 long, 0.76 wide, 0.7 high. Clypeus 0.08 high. Sternum 0.3 long, 0.32 wide. Length of legs: I 1.46 (0.50, 0.16, 0.40, 0.20, 0.20); II 1.32 (0.40, 0.20, 0.30, 0.20, 0.22); III 0.88 (0.30, 0.10, 0.20, 0.16, 0.12); IV 1.04 (0.36, 0.10, 0.24, 0.16, 0.18).

**Figure 10.**
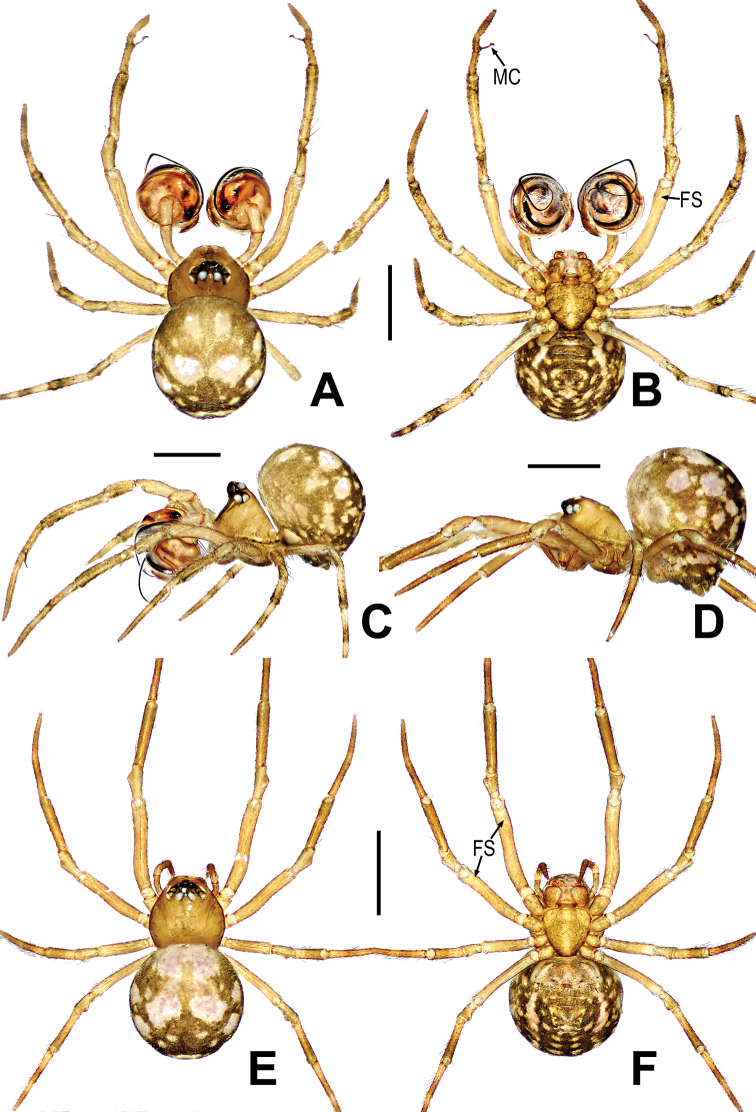
*Mosuheguomu* sp. nov. **A–C** male habitus **D–F** female habitus **A, E** dorsal **B, F** ventra **C, D** lateral. Abbreviations: FS = femoral spot; MC = Metatarsal clasping spine. Scale bars: 0.50 mm.

***Somatic characters*** (Fig. 10A–C, 11D). ***Coloration***: prosoma brown-yellow. Ocular base black. Chelicera yellow. Endites and labium light-yellow. Sternum brown marginally yellow centrally. Legs yellowish brown. Abdomen dark brown with multiple symmetric light-yellow spots. ***Prosoma***: carapace pentagon-shaped in dorsal and peak shape in lateral, marginally not smooth. Cephalic area elevated. Ocular region projecting, eight eyes in two rows. AER and PER recurved in dorsal view, ALE and PLE contiguous. Labium rectangular. Sternum scutiform, covered with short setae. ***Legs***: leg I with a mating clasper on metatarsus, and with a subdistal sclerotized femoral spot at surface of ventral femur. Legs covered with setae and bristles. ***Abdomen***: round in dorsum, covered with pale short setae.

**Figure 11.**
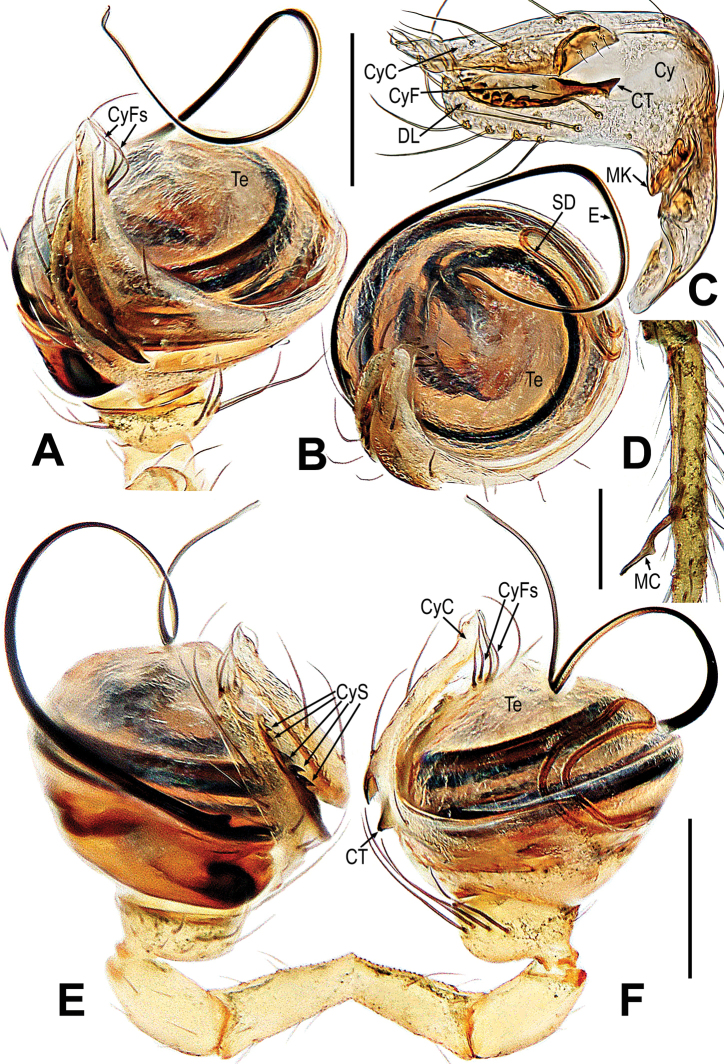
*Mosuheguomu* sp. nov. **A, B, E, F** male palp **C** cymbium **D** male left metatarsus I **A, C** ventral **B** apical **D, E** prolateral **F** retrolateral. Abbreviations: CT = cymbial tooth; Cy = cymbium; CyC = cymbial conductor; CyF = cymbial fold; CyFs = setae on cymbial fold; CyS = cymbial serrula; DL = distal lobe on cymbium; E = embolus; MC = Metatarsal clasping spine; MK = median keel on cymbium; SD = spermatic duct; Te = tegulum; Ti = palpal tibia. Scale bars: 0.20 mm (**A–C, E, F**); 0.10 mm (**D**).

***Palp*** (Fig. 11A–C, E, F): orange; tibia small, about 1/5 volume of the bulb, with a row of long setae almost encircling the brim (Fig. 11E, F). Cymbium nearly transparent, “right angle”-shaped, with a cymbial tooth at the ventral median, a list of cymbial serrula, a distal lobe and a median keel on cymbium; the cymbial fold slightly sclerotized, with a row of setae; the tip of cymbium specialized as cymbial conductor (Fig. 11A, C, E). Bulb oblate, embedded in a translucent membranous tegulum. Embolus filiform, length of embolus coiled into two loops in tegulum, the apical of embolus coiled on the bulb (Fig. 11A, B, E, F).

**Female. *Measurements***: total length 1.14. Prosoma 0.4 long, 0.48 wide, 0.4 high. Abdomen 0.7 long, 0.7 wide, 0.9 high. Clypeus 0.06 high. Sternum 0.3 long, 0.28 wide. Length of legs: I 1.16 (0.50, 0.12, 0.14, 0.22, 0.18); II 1.06 (0.44, 0.12, 0.14, 0.20, 0.16); III 0.92 (0.28, 0.08, 0.20, 0.16, 0.20); IV 1.22 (0.40, 0.10, 0.30, 0.24, 0.18).

***Somatic characters*** (Fig. 10D–F). ***Coloration***: prosoma, chelicera endites, labium and sternum yellow-brown. Ocular base black. Legs yellow. Abdomen dark brown with light yellow spots. ***Prosoma***: carapace near pear-shaped, marginally not smooth. Cephalic part slightly elevated. Eight eyes in two rows, white, with black rings. ALE and PLE contiguous. Labium triangle. Sternum scutiform, plump, covered with sparse setae. ***Legs***: a sclerotized femoral spot present at apical ventral surface of leg I and II; covered with setae and bristles. ***Abdomen***: round in dorsum.

**Figure 12.**
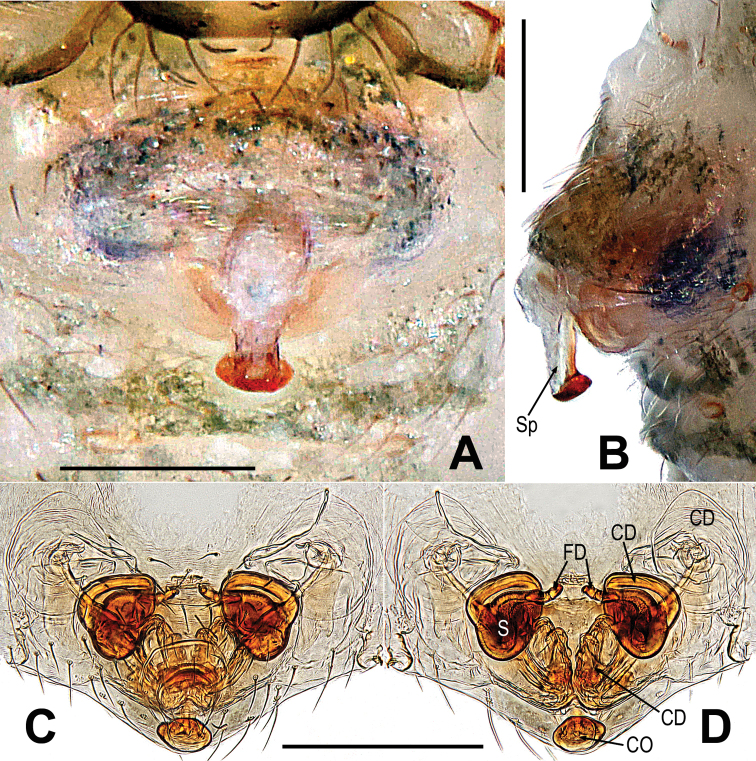
*Mosuheguomu* sp. nov. **A, B** epigyne **C, D** vulva **A, C** ventral **B** lateral **D** dorsal. Abbreviations: CD = copulatory duct; CO = copulatory opening; FD = fertilization duct; S = spermatheca; Sp = scape. Scale bars: 0.10 mm.

***Epigyne*** (Fig. 12A–D): scape long, the tip with a sclerotized and expanded copulatory opening (Fig. 12A–D). Spermathecae oval, inclined at 45 degrees. Fertilization ducts short, derived from anterior border of spermathecae. Copulatory ducts around the spermathecae, coiled into multiple loops below the spermathecae (Fig. 12C, D).

##### Distribution.

Southwestern China (Yunnan).

##### Remarks.

The genus *Mosu* established by Miller et al. (2009) based on only known females of two species (*M.nujiang* and *M.huogou*), the common characteristics of the genus: kidney-shaped spermathecae, sclerotized fertilization ducts, the copulatory duct membranous and convoluted, sclerotized at end of path near spermathecae. Lin et al. (2013) supplemented male morphological characters, which distinguish the male palps: cymbial process present; bulb nearly globose; tegulum plump, lacks movable sclerites; embolus long, filiform, coiling into two loops under tegulum and reaching to the distal end of cymbium. This new species conforms to this generic characters, but can be clearly distinguished from *M.dayan* Lin & Li, 2013, *M.huogou* Miller, Griswold & Yin, 2009, *M.nujiang* Miller, Griswold & Yin, 2009, *M.tanjia* Lin & Li, 2013, we propose it as a new species.

#### ﻿ Mosu zhengi

Taxon classificationAnimaliaAraneaeMysmenidae

(Lin & Li, 2008) comb. nov.

4F215F6D-9468-52C8-9520-7CC416BDAF9F

Figs 13 , 14 , 15

Mysmena zhengi Lin and Li 2008: 490, figs 3A–E, 4A–H (♂♀).

##### Type material.

***Holotype*** ♂ (IZCAS) and ***paratypes*** 6♂ 6♀, China: Yunnan, Mengla, Menglun, Primary tropical seasonal rainforest in XTBG (21.917°N, 101.275°E; 558±17 m), by pitfall trapping, 22.VII.2007, G. Zheng leg. Examined.

##### Other material examined.

2♂ 3♀ (NHMSU), China: Yunnan, Mengla, Menglun, XTBG, *Paramicheliabaillonii* plantation (about 20 yr.) (21.903°N, 101.282°E; 608±11 m), by pitfall trapping, 7–11.VIII.2006, G. Zheng leg.; 1♂ 1♀ (NHMSU), China: Yunnan, Mengla, Menglun, XTBG, *Paramicheliabaillonii* plantation (about 20 yr.) (21.913°N, 101.267°E; 556±11 m), by searching, 19–26.XI.2006, G. Zheng leg.; 1♀ (IZCAS), China: Yunnan, Mengla, Menglun, XTBG, Rubber-Tea Plantation (about 20 yr.) (21.908°N, 101.266°E; 569±11 m), by searching, 5–12.III.2007, G. Zheng leg.

##### Diagnosis.

**Figure 13.**
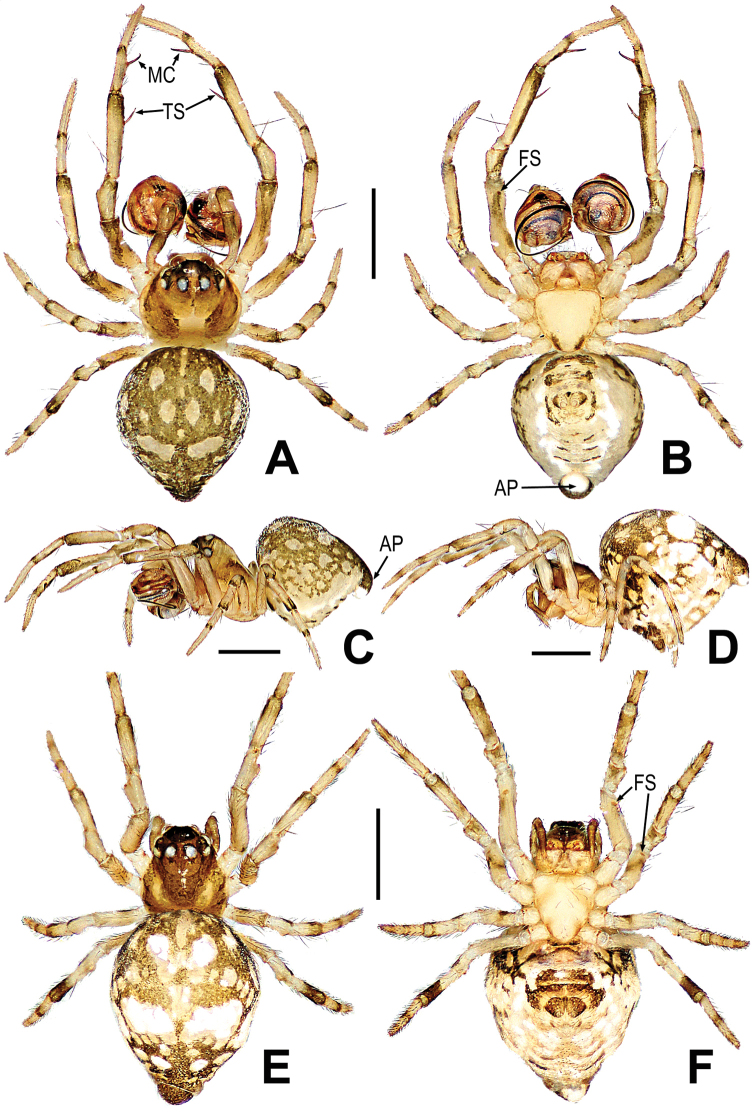
*Mosuzhengi* comb. nov. **A–C** male habitus **D–F** female habitus **A, E** dorsal **B, F** ventral **C, D** lateral. Abbreviations: AP = abdominal protuberance; FS = femoral spot; MC = Metatarsal clasping spine; TS = tibial spine on male leg I. Scale bars: 0.50 mm.

**Figure 14.**
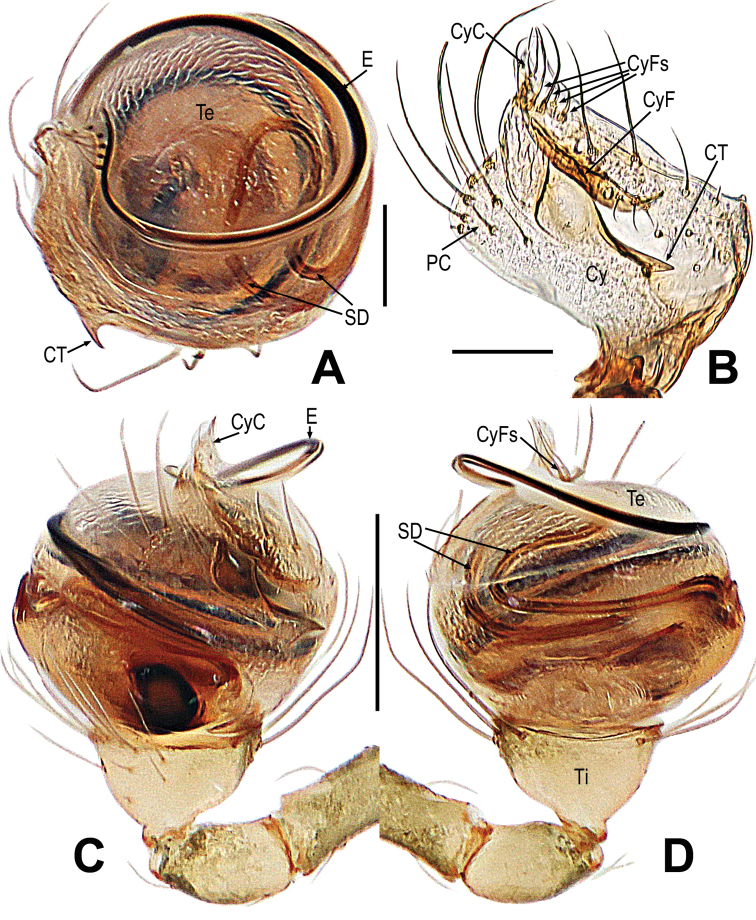
*Mosuzhengi* comb. nov. **A, C, D** male palp **B** cymbium. **A** apical **B** proventral **C** prolateral **D** retrolateral. Abbreviations: Abbreviations: CT = cymbial tooth; Cy = cymbium; CyC = cymbial conductor; CyF = cymbial fold; CyFs = setae on cymbial fold; E = embolus; PC = paracymbium; SD = spermatic duct; Te = tegulum; Ti = palpal tibia. Scale bars: 0.10 mm (**A, B**); 0.20 mm (**C, D**).

**Figure 15.**
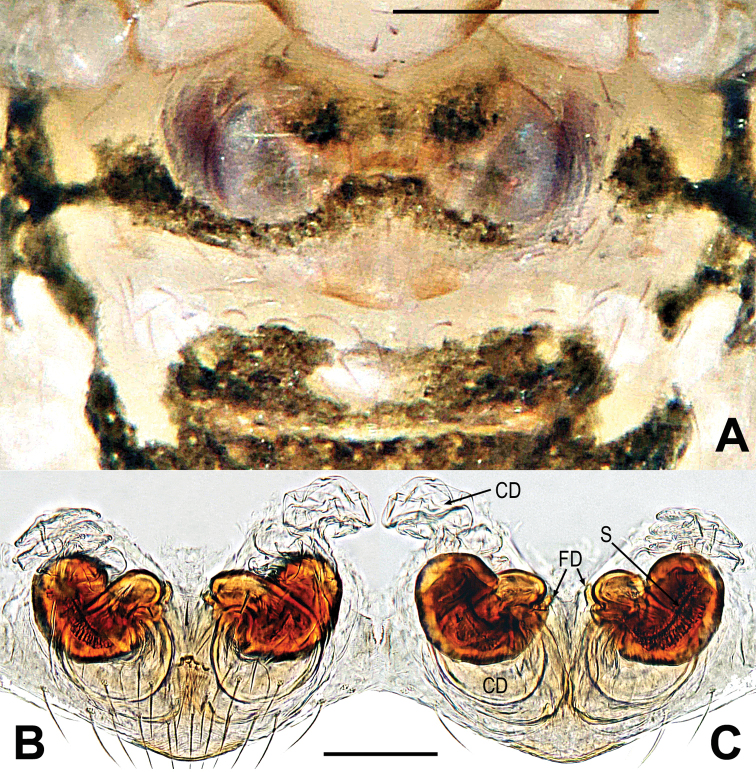
*Mosuzhengi* comb. nov. **A** epigyne **B–C** vulva. **A, B** ventral **C** dorsal. Abbreviations: CD = copulatory duct; FD = fertilization duct; S = spermatheca. Scale bars: 0.20 mm (**A**); 0.10 mm (**B, C**).

This species is similar to *M.tanjia* Lin & Li, 2013, but can be distinguished by the male and female each with a short abdominal protuberance, the male with sclerotized femoral spot present on the surface of ventral femur I, the female with a femoral spot present on the surfaces of femur I and II (Fig. 13A–F vs. fig. 7A–F, Lin et al. 2013: 458). The palp can be distinguished by the cymbial tooth located in the cymbial center (Fig. 14B–C vs. figs 8A, C, 10C, Lin et al. 2013: 459). The female can be distinguished by the margin inferior to the epigyne incrassate, the reniform spermathecae, and the copulatory ducts without a curve and sclerotized parts above the spermathecae (Fig. 15A–C vs. figs 9A, B, 12A, B, Lin et al. 2013: 460, 463).

##### Description.

**Male. *Measurements***: total length 1.45. Prosoma 0.55 long, 0.55 wide, 0.43 high. Abdomen 0.90 long, 0.75 wide, 0.75 high. Clypeus 0.08 high. Sternum 0.38 long, 0.35 wide. Length of legs: I 1.44 (0.43, 0.18, 0.38, 0.20, 0.25); II 1.19 (0.38, 0.13, 0.25, 0.18, 0.25); III 0.69 (0.25, 0.10, 0.10, 0.12, 0.12); IV 0.98 (0.33, 0.10, 0.30, 0.15, 0.10).

***Somatic characters*** (Fig. 13A–C). ***Coloration***: prosoma deep yellow dorsally, yellow ventrally, ocular base black. Abdomen brown, with multiple yellow spots. Legs brown-yellow. ***Prosoma***: carapace near round in dorsal and peak-shaped in lateral, marginally smooth. Cephalic area sharply elevated. Ocular region projecting, eight eyes in two rows. All eyes round, AER and PER recurved in dorsal view, ALE and PLE contiguous. Labium rectangle. Sternum scutiform, smooth surface. ***Legs***: leg I with a mating clasper on metatarsus, a subdistal sclerotized femoral spot present at surface of ventral femur, the two spines on tibia. Legs covered with setae and bristles. ***Abdomen***: near ladle-shaped in dorsum, covered with pale short setae.

***Palp*** (Fig. 14A–D): orange; tibia cup-shaped, except for retrolateral region, a row of long setae almost encircled the distal brim (Fig. 14C, D). Cymbium transparent, nearly slant parallelogram, with a thorn-shaped cymbial tooth, cymbial fold long and sclerotized, bears a row of ordered setae (Fig. 14A, B); Cymbial conductor wide, arc (Fig. 14B). Paracymbium with long setae (Fig. 14B). Bulb near round, embedded in a translucent membranous tegulum. Embolus long, coiled into 2 loops (Fig. 14A, C, D).

**Female. *Measurements***: total length 1.58. Prosoma 0.45 long, 0.45 wide, 0.30 high. Abdomen 1.13 long, 0.85 wide, 0.80 high. Clypeus 0.08 high. Sternum 0.38 long, 0.32 wide. Length of legs: I 1.10 (0.35, 0.10, 0.25, 0.20, 0.20); II 1.01 (0.25, 0.10, 0.24, 0.22, 0.20); III 0.76 (0.23, 0.08, 0.20, 0.15, 0.10); IV 0.92 (0.20, 0.15, 0.25, 0.16 0.16).

***Somatic characters*** (Fig. 13D–F). ***Coloration***: prosoma deep yellow dorsally, yellow ventrally, ocular base black. Abdomen brown-yellow, with multiple white spots. Legs brown-yellow. ***Prosoma***: carapace long, nearly pear-shaped. Cephalic part lower than in male, flatted on top. Eight eyes in three rows. AER and PER straight in dorsal view. Chelicerae, endites as in male, labium triangle, and sternum scutiform. ***Legs***: covered with setae and bristles, a sclerotized subdistal-ventral femoral spot present at surface of leg I and II. ***Abdomen***: same as in male.

***Epigyne*** (Fig. 15A–C): spermathecae big, reniform (Fig. 15B–C). Fertilization ducts short, derived from anterior border of spermathecae. Copulatory ducts membranous, slightly sclerotized, around the spermathecae; the part of below the spermathecae coiled into two loops (Fig. 15C).

##### Distribution.

Southwestern China (Yunnan).

##### Remarks.

Miller et al. (2009) established the genus *Mosu* based on only known females of two species (*M.jujiang* and *M.huogou*), while studying the symphytognathoid spiders of the Gaoligongshan Mountain. They thought that *Mysmenazhengi* Lin & Li, 2008 may also belongs to this genus (The species consistent with the common characteristics of the genus: reniform and sclerotized spermathecae, sclerotized fertilization ducts, the copulatory duct membranous and convoluted, sclerotized at end of path near spermathecae). In this paper, we formally proposed transferring this species to *Mosu* as a new combination, based on a similar configuration of the vulva.

### *Mysmena* Simon, 1894

#### ﻿ Mysmena arcilonga

Taxon classificationAnimaliaAraneaeMysmenidae

Lin & Li, 2008

7F7C60E9-6D3C-599A-A1FA-BBCC36F757A4

Figs 16 , 17 , 18

Mysmena arcilongus Lin and Li 2008: 497, fig. 7A–I (♂).

##### Type material.

***Holotype*** ♂ (IZCAS), China: Yunnan, Mengla, Menglun, XTBG, Rubber plantation (21.908°N, 101.266°E; 569±11 m), by searching, 21.VII.2007, G. Zheng leg. Examined.

##### Other material examined.

8♂ 25♀ (IZCAS), China: Yunnan, Mengla, Menglun, XTBG, primary tropical seasonal rainforest (21.926°N, 101.406°E; 558±17 m), by searching, 5–12.IX.2006, G. Zheng leg.; 3♂ 2♀ (NHMSU), China: same site as for preceding (22.136°N, 101.431°E; 790±15 m), by searching, 5–12.I.2007, G. Zheng leg.

##### Diagnosis.

**Figure 16.**
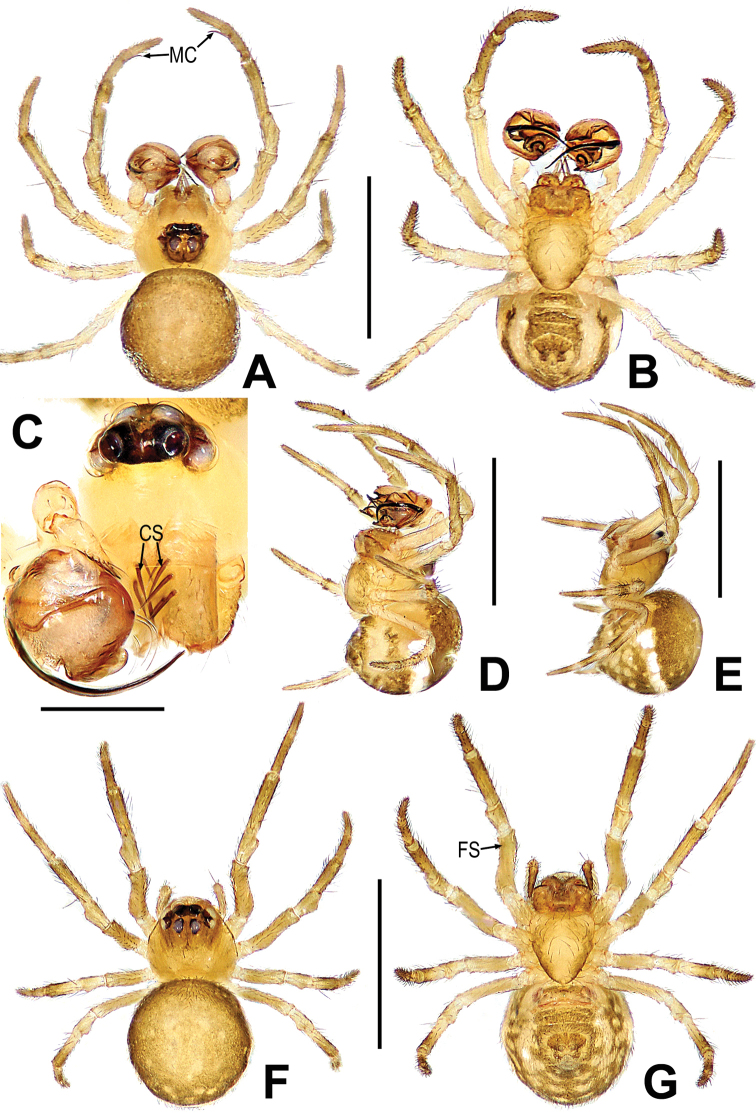
*Mysmenaarcilonga***A, B, D** male habitus **C** male prosoma **E–G** female habitus **A, F** dorsal **B, G** ventral **C** anterolateral **D, E** lateral. Abbreviations: CS = cheliceral spines on male; FS = femoral spot; MC = Metatarsal clasping spine. Scale bars: 0.50 mm (**A, B, D–G**); 0.20 mm (**C**).

**Figure 17.**
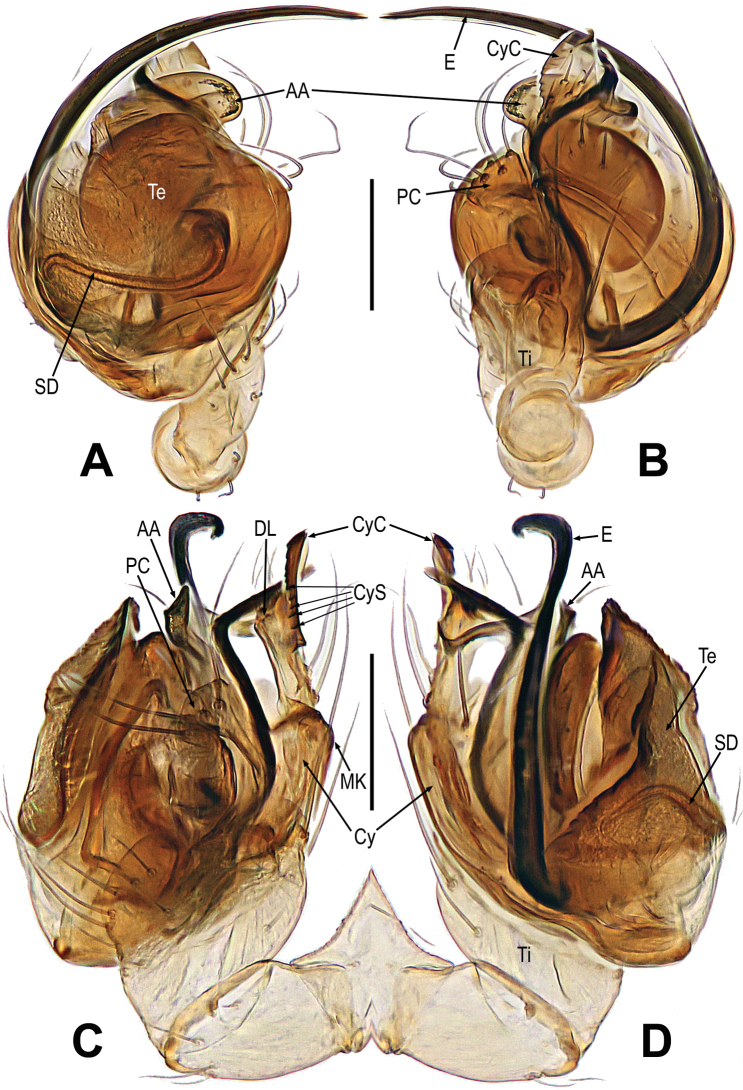
*Mysmenaarcilonga***A–D** male palp **A** dorsal **B** ventral **C** prolateral **D** retrolateral. Abbreviations: AA = apical apophysis on tegulum; Cy = cymbium; CyC = cymbial conductor; CyS = cymbial serrula; DL = distal lobe on cymbium; E = embolus; MK = median keel on cymbium; PC = paracymbium; SD = spermatic duct; Te = tegulum; Ti = palpal tibia. Scale bars: 0.10 mm.

**Figure 18.**
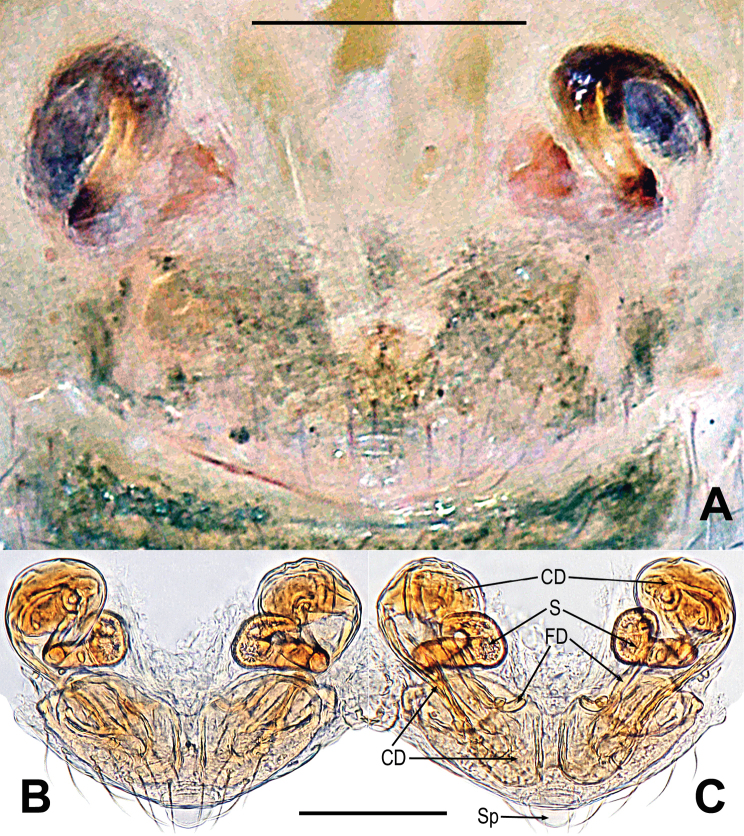
*Mysmenaarcilonga***A** epigyne **B–C** vulva **A, B** ventral **C** dorsal. Abbreviations: CD = copulatory duct; FD = fertilization duct; S = spermatheca; Sp = scape. Scale bars: 0.10 mm (**A–C**); 0.20 mm (**A**); 0.10 mm (**B–C**).

This species can be distinguished from other congeners except for *M.furca*, *M.luosuo* sp. nov., and *M.rostella* by the presence of modified cheliceral spines on males, a row of cymbial serrula on the cymbium, a long, bow-shaped embolus spans retrolaterally to the entire bulbus, and the partial swollen copulatory ducts larger than the spermathecea (cf. Figs 16C, 17A–D, 18B–C). Its males differed from that of *Mysmenafurca*, *M.luosuo* sp. nov., and *M.rostella* by having a long, bow-shaped embolus and a serrated cymbial conductor (CyC, Fig. 17B, C), but short embolus in *M.furca* (Fig. 23C), twisted embolus and absence of a serrated CyC in *M.luosuo* sp. nov. (Fig. 25B, E), long hooked embolus and CyC with a distal keel in *M.rostella* (Fig. 28A, C). Females by the curved, rod-shaped spermathecae and the long fertilization ducts (Fig. 18C), but transverse ovoid spermathecae and short fertilization ducts in *M.furca* and *M.luosuo* sp. nov. (Figs 23F, 26C), reniform spermathecae in *M.rostella* (Fig. 29C).

##### Description.

**Male.** See Fig. 16A–D and Lin and Li (2008): 497.

***Palp*** (Fig. 17A–D): Orange, the tibia comparatively small, about one-quarter the volume of the bulb; except for retrolateral region, a row of long setae almost encircled the distal brim of tibia (Fig. 17A–D). Cymbium nearly transparent, the tip specialized as a wide cymbial conductor; a row of cymbial serrula on the cymbium; there is a distal lobe on cymbium and a median keel on the middle of the cymbium (Fig. 17B–D). Paracymbium big, with long setae (Fig. 17B–C). Tegulum translucent membranous, with apical apophysis. Embolus long, with two ends, one end extends to cymbial conductor, the other end extends upon the tegulum (Fig. 17A–D).

##### New morphological data.

**Female. *Measurements***: total length 0.64 Prosoma 0.25 long, 0.27 wide, 0.16 high. Abdomen 0.39 long, 0.39 wide, 0.32 high. Clypeus 0.05 high. Sternum 0.23 long, 0.18 wide. Length of legs: I 0.70 (0.19, 0.08, 0.16, 0.13, 0.14); II 0.67 (0.13, 0.08, 0.18, 0.14, 0.14); III 0.46 (0.11, 0.07, 0.12, 0.08, 0.08); IV 0.53 (0.16, 0.10, 0.13, 0.08 0.06).

***Somatic characters*** (Fig. 16E–G). ***Coloration***: same as in male. ***Prosoma***: carapace nearly peach-shaped. Ocular region projecting, eight eyes in two rows, ALE and PLE contiguous. Chelicerae, endites and labium as in male, the sternum scutiform, covers with short setae. ***Legs***: covered with setae and bristles, a sclerotized subdistal-ventral femoral spot present at surface of leg I. ***Abdomen***: same as in male.

***Epigyne*** (Fig. 18A–C): the scape short, surface with sparse fold (Fig. 18B). Spermathecae small, irregular (Fig. 18B–C). Fertilization ducts long, derived from anterior border of spermathecae and extended posteriorly. Copulatory ducts long and membranous, the other part slightly sclerotized, extending anteriorly to form an oval (Fig. 18C).

##### Distribution.

Southwestern China (Yunnan).

##### Remarks.

The female of *M.arcilonga* is reported for the first time.

#### ﻿ Mysmena biangulata

Taxon classificationAnimaliaAraneaeMysmenidae

(Lin & Li, 2008)

5B595A91-F062-5C57-BFC3-7C7FCEB829AA

Figs 19 , 20

Calodipoena biangulata Lin and Li 2008: 499, figs 8A–E, 9A–H (♂♀).
Mysmena biangulata Lopardo and Hormiga 2015: 784.

##### Type material.

***Holotype*** ♂ (IZCAS) and ***paratypes*** 10♂ 7♀ (IZCAS), China: Yunnan, Mengla, XTBG, secondary tropical seasonal rainforest (21.924°N, 101.274°E; 598±17 m), by pitfall trapping, 22.VII.2007, G. Zheng leg. Examined.

##### Other material examined.

19♂ 27♀ (IZCAS), China: Yunnan, Mengla, Menglun, XTBG, Rubber-Tea plantation (about 20 yr.) (21.908°N, 101.266°E; 569±11 m), by pitfall trapping, 16–31.I.2007, G. Zheng leg.; 3♂ 10♀ (NHMSU), China: Yunnan, Mengla, Menglun, XTBG, *Paramicheliabaillonii* plantation (about 20 yr.) (21.897°N, 101.285°E; 613±11 m), by pitfall trapping, 16–24.X.2006, G. Zheng leg.

##### Diagnosis.

This species can be distinguished from other species except for *M.awari* (Baert, 1984), *M.marijkeae* (Baert, 1982), *M.vangoethemi* (Baert, 1982) and *M.nubiai* (Baert, 1984) by the elongate palpal bulbus, the cymbial process (CyP) juxtaposed with cymbial conductor (CyC) and both curved (cf. Fig. 20B, figs 11–12 in Baert 1982, and figs 9–10, 12–13 in Baert 1984), and the twisted, widely spaced spermathecae (cf. Fig. 20E, fig. 9H in Lin and Li 2008). *Mysmenabiangulata* distinguished from those four species by CyP near same length as CyC at *M.biangulata*, shorter in four species (Fig. 20A, B vs. fig. 133D–F, Lopardo & Hormiga, 2015, 676, figs 11, 12, Baert, 1982, 306, figs 9–11, Baert, 1984b, 231). One CyP in *M.biangulata*, two processes in *M.awari* (fig. 133D–E in Lopardo & Hormiga, 2015), *M.vangoethemi* (fig. 12 in Baert, 1982) and *M.nubiai* (figs 9–11, in Baert, 1984), three processes in *M.marijkeae* (fig. 11 in Baert 1982). Females can be distinguished by the coiled spermathecae with modified glandulous sac and the directly opposite basal partition of copulatory ducts (Fig. 20D, E).

**Figure 19.**
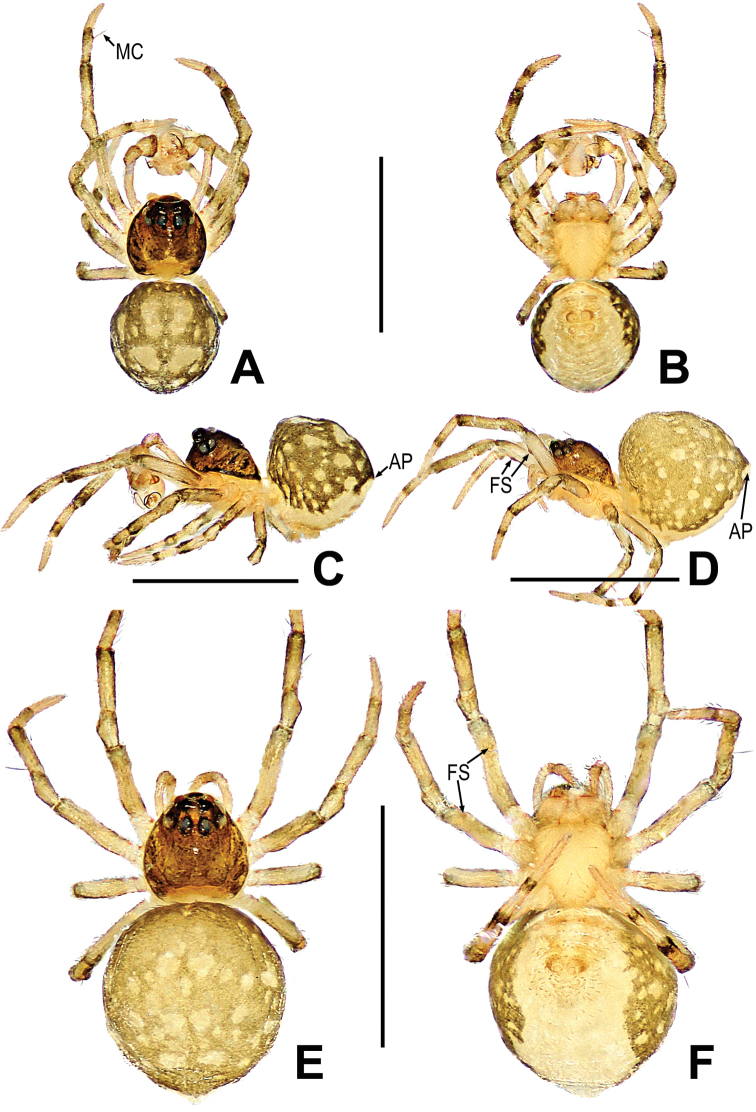
*Mysmenabiangulata***A–C** male habitus **D–F** female habitus **A, E** dorsal **B, F** ventral **C, D** lateral. Abbreviations: AP = abdominal protuberance; FS = femoral spot; MC = Metatarsal clasping spine. Scale bars: 0.50 mm.

##### Description.

See Fig. 19A–F and Lin and Li 2008: 499.

**Figure 20.**
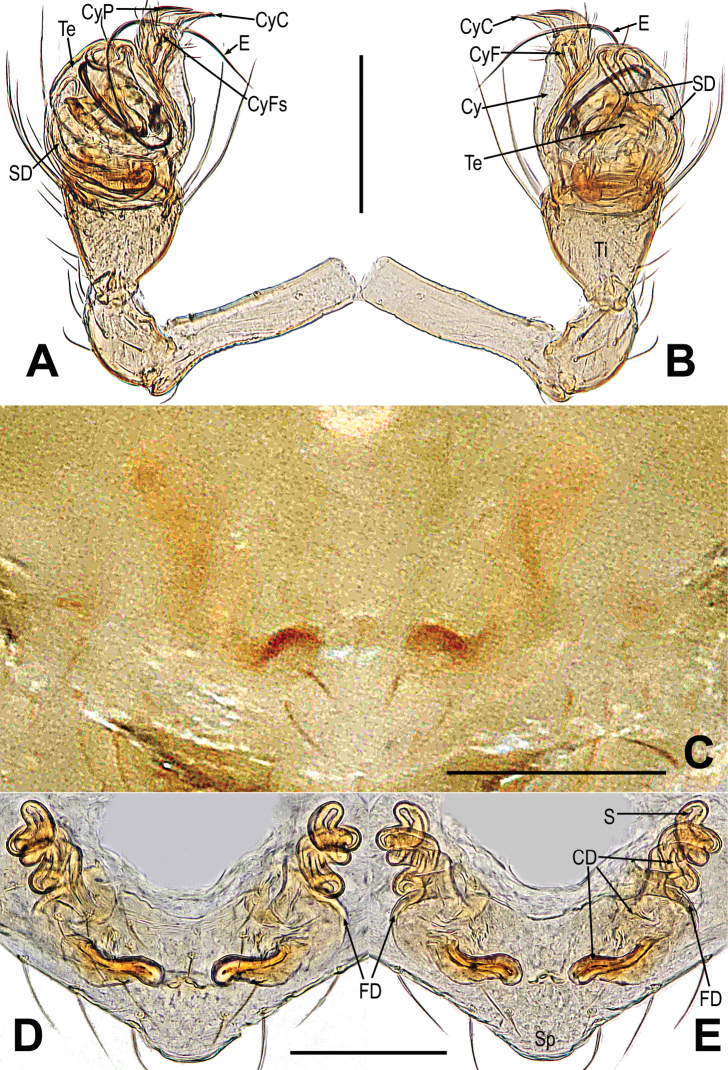
*Mysmenabiangulata***A, B** male palp **C** epigyne **D, E** vulva. **A** prolateral **B** retrolateral **C, D** ventral **E** dorsal. Abbreviations: CD = copulatory duct; Cy = cymbium; CyC = cymbial conductor; CyF = cymbial fold; CyFs = setae on cymbial fold; CyP = cymbial process; E = embolus; FD = fertilization duct; S = spermatheca; SD = spermatic duct; Sp = scape; Te = tegulum; Ti = palpal tibia. Scale bars: 0.20 mm (**A, B**); 0.10 mm (**C–E**).

***Male palp*** (Fig. 20A, B): light-yellow; tibia big, about 2/3 volume of the bulb, cup-shaped; Except for retrolateral region, a row of long setae almost encircling the distal brim (Fig. 20B). Cymbium nearly transparent; the cymbial conductor lateral bending, parallel to the cymbial process; the cymbial fold long and sclerotized, bears a row of ordered setae (Fig. 20A, B); Embolus threadlike, coiled into 2 loops in tegulum. Spermatic ducts can be seen through tegulum (Fig. 20A, B).

***Epigyne*** (Fig. 20C–E). The scape stubby, surface smooth (Fig. 20C–E). Spermathecae small, the diameter same as the copulatory ducts (Fig. 20D). Fertilization ducts and copulatory ducts slightly sclerotized, coiling around each other; the fertilization ducts opening to both edges of epigyne; anterior copulatory ducts sclerotized, flow-shaped; two openings converge toward the centre of epigyne (Fig. 20D, E).

##### Distribution.

Southwestern China (Yunnan).

#### ﻿ Mysmena cornigera

Taxon classificationAnimaliaAraneaeMysmenidae

(Lin & Li, 2008)

BDA4B805-DADC-58A0-BA7D-D5E8B74B5159

Fig. 21

Calodipoena cornigera Lin and Li 2008: 501, fig. 10A–J (♂).
Mysmena cornigera Lopardo and Hormiga 2015: 784.

##### Type material.

***Holotype*** ♂ (IZCAS), China: Yunnan, Mengla, XTBG, secondary tropical seasonal forest (21.907°N, 101.208°E; 612±11 m), by searching, 10.VIII.2007, G. Zheng leg. Examined.

##### Other material examined.

♂ (NHMSU), China: Yunnan, Mengla, Menglun, Baka Village Nature Reserve (21.722°N, 101.384°E; 716 m), by searching, 17.VIII.2011, Y. Lin leg.

##### Diagnosis.

**Figure 21.**
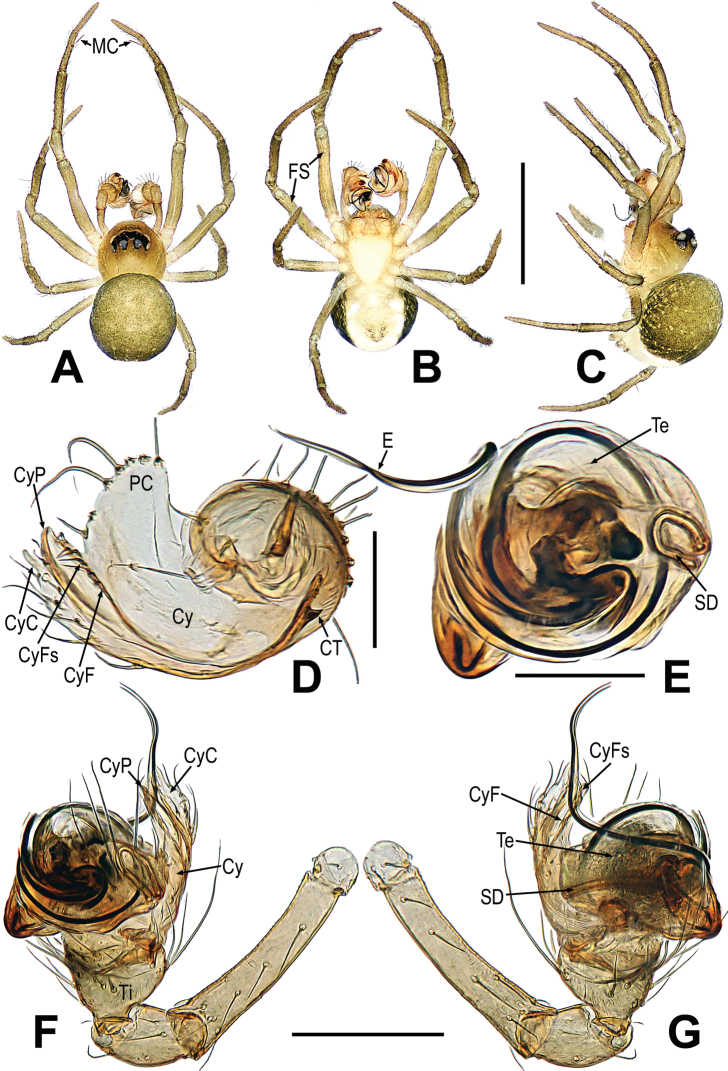
*Mysmenacornigera***A–C** male habitus **D** cymbium **E** bulbus **F, G** male palp **A** dorsal **B** ventral **C** lateral **D, E** apical **F** prolateral **G** retrolateral. Abbreviations: CT = cymbial tooth; Cy = cymbium; CyC = cymbial conductor; CyF = cymbial fold; CyFs = setae on cymbial fold; CyP = cymbial process; E = embolus; FS = femoral spot; MC= Metatarsal clasping spine; PC = paracymbium; SD = spermatic duct; Te = tegulum; Ti = palpal tibia. Scale bars: 0.50 mm (**A–C**); 0.10 mm (**D, E**); 0.20 mm (**F, G**).

This species seems close to *M.caribbaea* (Gertsch, 1960) and *M.stathamae* (Gertsch, 1960) in the shape of palpal bulbus, the earlobe-shaped paracymbium and the simple distal part of cymbium (cf. Fig. 21D–F, and figs 30–31, 35–36 in Gertsch 1960), but can be distinguished by lacking a posterior abdominal tubercle, having a cymbial tooth and a distal process (CyP), (Fig. 21A, D, F and fig. 10A, B, G in Lin and Li 2008), with abdominal tubercle and lacking cymbial tooth and CyP in *M.caribbaea* and *M.stathamae* (figs 24, 27, 30–31, 35–36 in Gertsch, 1960).

##### Description.

**Male**. See Fig. 21A–C and Lin and Li (2008): 501.

***Palp*** (Fig. 21D–G): light-orange, comparatively large; tibia cup-shaped, except for retrolateral region, a row of long setae almost encircling the distal brim (Fig. 21F, G). Cymbium nearly transparent, the tip specialized as the cymbial conductor; cymbium with a process and a tooth-shaped cymbial tooth; cymbial fold long and slightly sclerotized, bore a row of ordered setae; paracymbium large, with long setae (Fig. 21D). Embolus threadlike, coiled into 1.5 loops in tegulum. Tegulum nearly transparent. Spermatic ducts can be seen through tegulum (Fig. 21F, G).

**Female**. Unknown.

##### Distribution.

Southwestern China (Yunnan).

#### ﻿ Mysmena furca

Taxon classificationAnimaliaAraneaeMysmenidae

Lin & Li, 2008

25356883-C704-5F24-BF62-09B35A1AF70A

Figs 22 , 23

Mysmena furca Lin & Li, 2008: 495, fig. 6A–G (♂).

##### Type material.

***Holotype*** ♂ (IZCAS), China: Yunnan, Mengla, Menglun, XTBG, Rubber plantation (21.908°N, 101.266°E; 569±11 m), by searching, 21.VII.2007, G. Zheng leg. Examined.

##### Other material examined.

22♂ 16♀ (IZCAS), China: Yunnan, Mengla, Menglun, XTBG, secondary tropical seasonal rainforest (21.924°N, 101.274°E; 598±17 m), by pitfall trapping, 16–31.III.2007, G. Zheng leg.; 5♂ 14♀ (NHMSU), China: Yunnan, Mengla, Menglun, XTBG, primary tropical seasonal rainforest (21.917°N, 101.275°E; 558±17 m), by searching, 4–11.IV.2007, G. Zheng leg.

##### Diagnosis.

**Figure 22.**
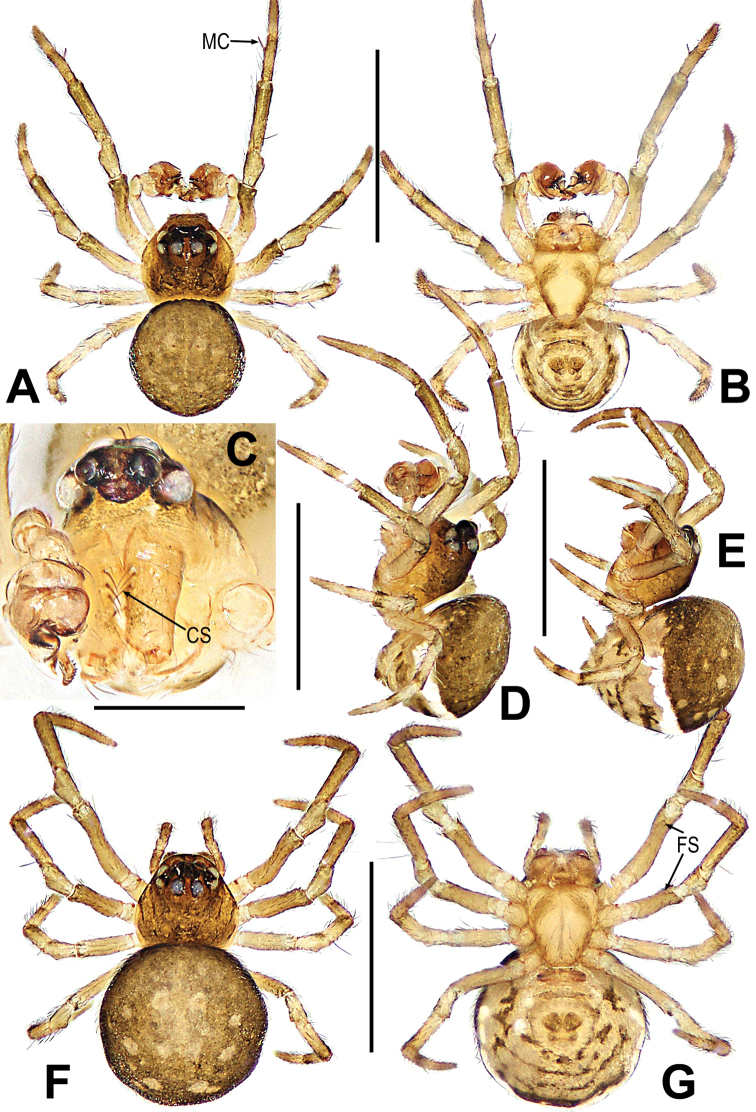
*Mysmenafurca***A, B, D** male habitus **C** male prosoma **E–G** female habitus **A, F** dorsal **B, G** ventral **C** anterolateral **D, E** lateral. Abbreviations: CS = cheliceral spines on male; FS = femoral spot; MC = Metatarsal clasping spine. Scale bars: 0.50 mm (**A, B, D–G**); 0.20 mm (**C**).

**Figure 23.**
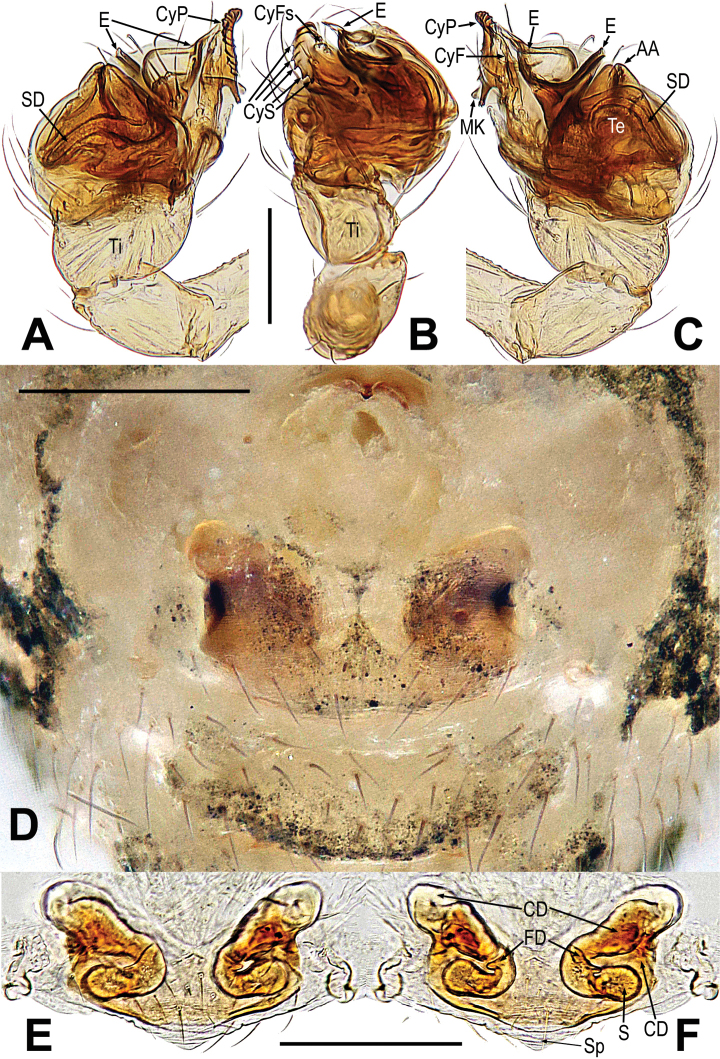
*Mysmenafurca***A–C** male palp **D** epigyne **E, F** vulva. **A** prolateral **B, D, E** ventral **C** retrolateral **F** dorsal. Abbreviations: AA = apical apophysis on tegulum; CD = copulatory duct; Cy = cymbium; CyC = cymbial conductor; CyF = cymbial fold; CyFs = setae on cymbial fold; CyP = cymbial process; CyS = cymbial serrula; E = embolus; FD = fertilization duct; MK = median keel on cymbium; S = spermatheca; SD = spermatic duct; Sp = scape; Te = tegulum; Ti = palpal tibia. Scale bars: 0.10 mm.

This species is similar to *M.arcilonga* but can be distinguished by the presence of four pairs of cheliceral spines (Fig. 22C vs. Fig. 16C), the palp presence of the cymbial fold, the cymbial process on the tip of cymbium; absence of distal lobe, a paracymbium and a cymbial conductor (Fig. 23A–C vs. Fig. 17A–D). The female can be distinguished by the spermathecae situated at the posterior of vulva, the diameter of copulatory ducts same as spermathecae, fertilization ducts shorter and extended to anterior of spermathecae (Fig. 23E, F vs. Fig. 18B, C).

##### Description.

**Male.** See Fig. 22A–D and Lin and Li (2008): 495.

***Palp*** (Fig. 23A–C): the tibia comparatively large, about the two-thirds volume of the bulb, except for retrolateral region, a row of long setae almost encircled the distal brim of tibia (Fig. 23A–C). Cymbium translucent, with a median keel and a row of cymbial serrula on the cymbium, the tip extended to be a cymbial process, and long cymbial fold slightly sclerotized, bears a row of short setae (Fig. 23A–C). The tegulum with apical apophysis, the embolus short, extended to cymbial conductor and the spermatic ducts can be seen through tegulum (Fig. 23A–C).

##### New morphological data.

**Female. *Measurements***: total length 0.70 Prosoma 0.26 long, 0.27 wide, 0.21 high. Abdomen 0.44 long, 0.44 wide, 0.38 high. Clypeus 0.06 high. Sternum 0.21 long, 0.13 wide. Length of legs: I 0.70 (0.24, 0.08, 0.20, 0.07, 0.11); II 0.64 (0.17, 0.08, 0.18, 0.10, 0.11); III 0.52 (0.16, 0.08, 0.12, 0.07, 0.09); IV 0.61 (0.21, 0.08, 0.12, 0.10 0.10).

***Somatic characters*** (Fig. 22E–G). ***Coloration***: same as in male. ***Prosoma***: carapace nearly peach-shaped. Ocular region projecting, eight eyes in two rows, ALE and PLE contiguous. Chelicerae, endites as in male, labium triangle, and sternum scutiform, covers with short setae. ***Legs***: covered with setae and bristles. A sclerotized subdistal-ventral femoral spot present at surface of leg I and II. ***Abdomen***: same as in male.

***Epigyne*** (Fig. 23D–F): The scape short, transparent, tip thin (Fig. 23F). spermathecae small, nearly round. Fertilization ducts short, derived from dorsal of spermathecae, and extended to anterior of spermathecae. Copulatory ducts sclerotized, the diameter of copulatory ducts same as spermathecae, connected to the lateral of spermathecae (Fig. 23E, F).

##### Distribution.

Southwestern China (Yunnan).

##### Remarks.

The female description of *M.furca* is provided for the first time.

#### ﻿ Mysmena luosuo

Taxon classificationAnimaliaAraneaeMysmenidae

Lin & Li sp. nov.

477BA7FF-EBAF-5A15-A73C-40DD37FDCBBF

https://zoobank.org/05B79993-3BFA-4F2A-9D10-DC195EED80B6

Figs 24 , 25 , 26

##### Type material.

***Holotype*** ♂ (IZCAS) and ***paratypes*** 10♂ 3♀ (IZCAS), China: Yunnan, Mengla, XTBG, secondary tropical seasonal moist forest (21.916°N, 101.283°E; 656±15 m), by pitfall trapping, 1–24.X.2007, G. Zheng leg.

##### Other material examined.

40♂ 6♀ (NHMSU), China: Yunnan, Mengla, Menlun Nature Reserve, secondary tropical seasonal moist forest (21.911°N, 101.283°E; 633±17 m), by pitfall trapping, 16–31.I.2007, G. Zheng leg.

##### Etymology.

The specific name derives from the Luosuo River, which is a main river in the type locality; noun in apposition.

##### Diagnosis.

**Figure 24.**
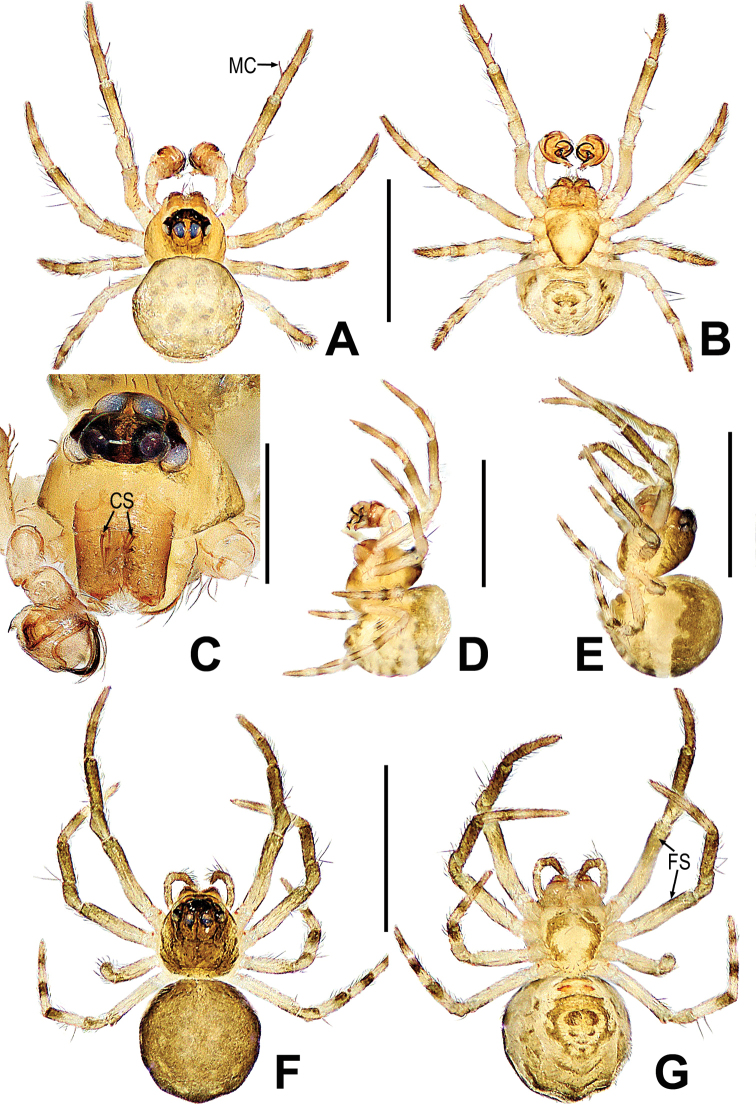
*Mysmenaluosuo* sp. nov. **A, B, D** male habitus **C** male prosoma **E–G** female habitus **A, F** dorsal **B, G** ventral **C** anterolateral **D, E** lateral. Abbreviations: CS = cheliceral spines on male; FS = femoral spot; MC = Metatarsal clasping spine. Scale bars: 0.50 mm (**A, B, D–G**); 0.20 mm (**C**).

**Figure 25.**
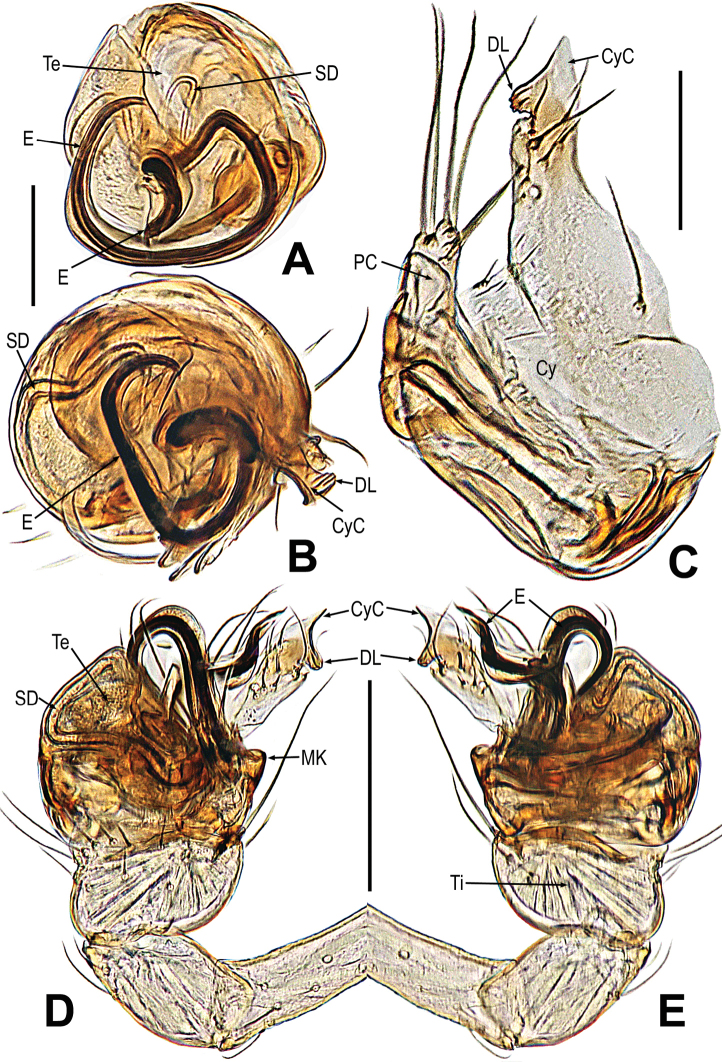
*Mysmenaluosuo* sp. nov. **A** bulbus **B** male palp **C** cymbium **D, E** male palp **A, B** apical **C** retroventral **D** prolateral **E** retrolateral. Abbreviations: Cy = cymbium; CyC = cymbial conductor; CyF = cymbial fold; CyFs = setae on cymbial fold; CyP = cymbial process; DL = distal lobe on cymbium; E = embolus; PC = paracymbium; MK = median keel on cymbium; SD = spermatic duct; Te = tegulum; Ti = palpal tibia. Scale bars: 0.10 mm (**A–C**); 0.20 mm (**D, E**).

**Figure 26.**
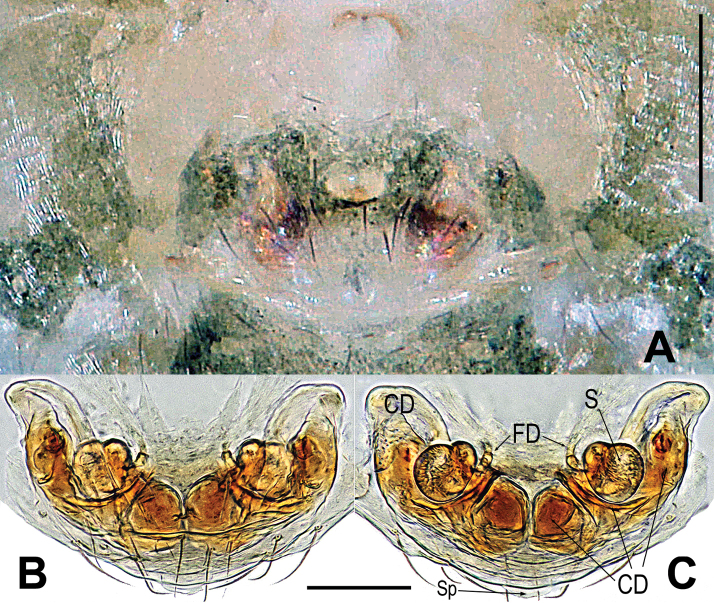
*Mysmenaluosuo* sp. nov. **A** epigyne **B–C** vulva **A, B** ventral **C** dorsal. Abbreviations: CD = copulatory duct; FD = fertilization duct; S = spermatheca; Sp = scape. Scale bars: 0.20 mm (**A**); 0.10 mm (**B–C**).

**Figure 27.**
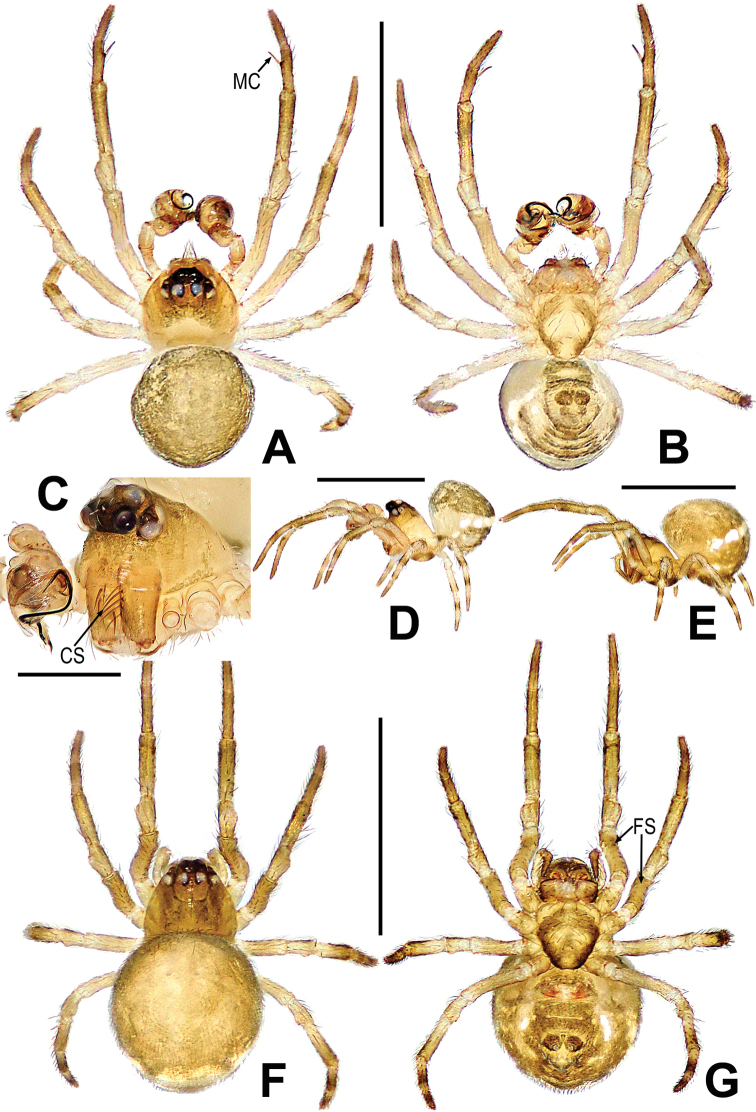
*Mysmenarostella***A, B, D** male habitus **C** male prosoma **E–G** female habitus **A, F** dorsal **B, G** ventral **C** anterolateral **D, E** lateral. Abbreviations: CS = cheliceral spines on male; FS = femoral spot; MC = Metatarsal clasping spine. Scale bars: 0.50 mm (**A, B, D–G**); 0.20 mm (**C**).

**Figure 28.**
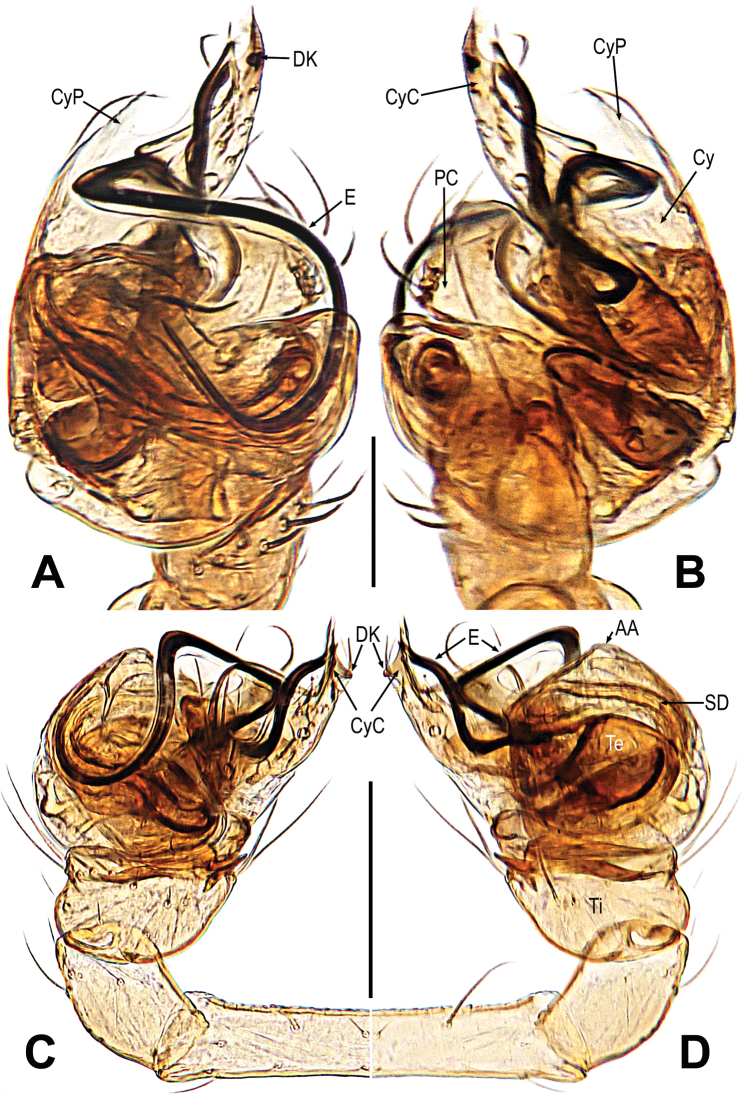
*Mysmenarostella***A–D** male palp **A** dorsal **B** ventral **C** prolateral **D** retrolateral. Abbreviations: AA = apical apophysis on tegulum; Cy = cymbium; CyC = cymbial conductor; CyP = cymbial process; DK = distal keel on cymbium; E = embolus; SD = spermatic duct; Te = tegulum; Ti = palpal tibia. Scale bars: 0.10 mm (**A, B**); 0.20 mm (**C, D**).

**Figure 29.**
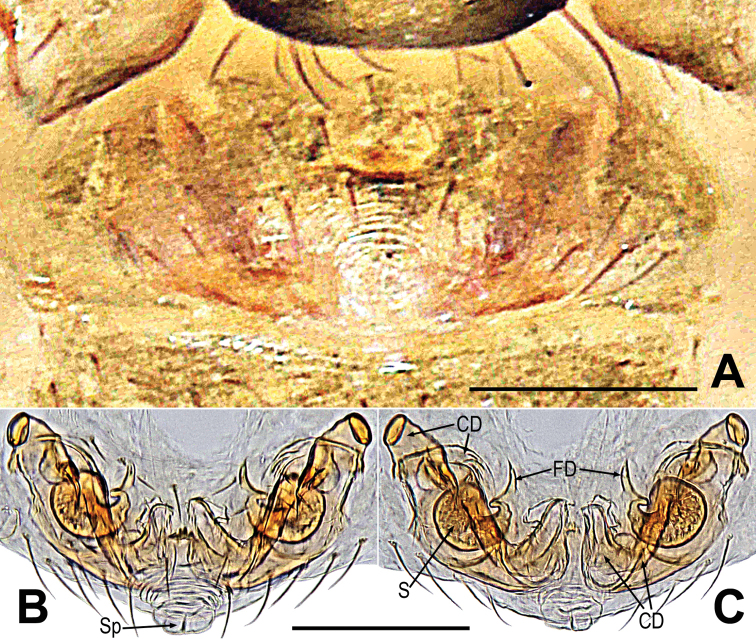
*Mysmenarostella***A** epigyne **B–C** vulva **A, B** ventral **C** dorsal. Abbreviations: CD = copulatory duct; FD = fertilization duct; S = spermatheca; Sp = scape. Scale bars: 0.10 mm (**A–C**).

*Mysmenaluosuo* sp. nov. seems similar to *M.furca* and *M.rostella* by the presence of modified cheliceral spines on the male (cf. Figs 22C, 24C, and 27C), the shape of the male palps (cf. Figs 25A, B, D, E and 28A–D), and the configuration of the vulvae (cf. Figs 26B, C, 23E, F). It can be distinguished from males of *M.furca* by lacking a serrated cymbial process (CyP), present in *M.furca* (Fig. 25C–E vs. Fig. 23A) and by a longer coiled embolus, shorter in *M.furca* (Fig. 25A, B vs. Fig. 23C); from *M.rostella* by the shorter embolus and lacking a cymbial process, but longer embolus and having cymbial process in *M.rostella* (Fig. 25C, D vs. Fig. 28A, B). Females can be distinguished from *M.furca* and *M.rostella* by the near globular spermathecae (Fig. 26C), ovoid in *M.furca* (Fig. 23F) and reniform in *M.rostella* (Fig. 29C).

##### Description.

**Male. *Measurements***: total length 0.57, Prosoma 0.21 long, 0.26 wide, 0.26 high. Abdomen 0.36 long, 0.37 wide, 0.40 high. Clypeus 0.06 high. Sternum 0.21 long, 0.20 wide. Length of legs: I 0.72 (0.21, 0.10, 0.18, 0.12, 0.11); II 0.68 (0.18, 0.10, 0.16, 0.10, 0.14); III 0.54 (0.12, 0.10, 0.12, 0.10, 0.10); IV 0.52 (0.14, 0.10, 0.12, 0.08, 0.08).

***Somatic characters*** (Fig. 24A–D). ***Coloration***: prosoma orange, with two brown spots ventrally, ocular base black. Abdomen yellow, with multiple light-brown spots. Legs brown-yellow. ***Prosoma***: carapace near pentagonal in dorsal. Cephalic area sharply elevated. Ocular region projecting, eight eyes in two rows. All eyes round, each eye surrounded by black ring. Three pairs of cheliceral spines. Labium nearly rectangular. Sternum scutiform, covered with sparse setae. ***Legs***: leg I with a mating clasper on metatarsus, covered with setae and bristles. ***Abdomen***: nearly round in dorsum, covered with pale short setae.

***Palp*** (Fig. 25A–E): orange, the tibia comparatively large, about half the volume of the bulb. Except for retrolateral region, a row of long setae almost encircled the distal brim of tibia (Fig. 25D, E). Cymbium nearly transparent, with a cymbial conductor, distal lobe and median keel on cymbium, the paracymbium comparatively small, with long setae (Fig. 25C–E). Bulb irregular, embedded in a translucent membranous tegulum. Spermatic ducts can be seen through tegulum. Embolus wide, coiled into “S”-shaped, tip extended to cymbial conductor (Fig. 25A–E).

**Female. *Measurements***: total length 0.64 Prosoma 0.26 long, 0.23 wide, 0.26 high. Abdomen 0.38 long, 0.35 wide, 0.38 high. Clypeus 0.05 high. Sternum 0.22 long, 0.20 wide. Length of legs: I 0.71 (0.28, 0.10, 0.13, 0.10, 0.10); II 0.64 (0.19, 0.10, 0.15, 0.10, 0.10); III 0.51 (0.15, 0.08, 0.08, 0.10, 0.10); IV 0.65 (0.23, 0.08, 0.12, 0.12 0.10).

***Somatic characters*** (Fig. 24E–G). ***Coloration***: prosoma brown dorsally, yellow ventrally with two brown strips, ocular base black. Abdomen brown dorsally, yellow ventrally with multiple arc brown strips and spots. Legs brown-yellow. ***Prosoma***: carapace nearly pear-shaped. The eight eyes in two rows, AER and PER recurved in dorsal view. ALE and PLE contiguous. Chelicerae, endites as in male, labium rectangle, and sternum scutiform, covers with short setae. ***Legs***: covered with setae and bristles. A sclerotized subdistal-ventral femoral spot present at surface of leg I and II. ***Abdomen***: nearly round in dorsum, covered with short brown setae.

***Epigyne*** (Fig. 26A–C): The scape short, transparent (Fig. 26C). The spermathecae globular, situated at the middle of vulva. Fertilization ducts short, derived from dorsal of the spermathecae and coiled to anterior of spermathecae. Copulatory ducts sclerotized and wider, coiled around the spermathecae, the posterior part expanded to a globular, connected to the ventral of spermathecae. (Fig. 26B, C).

##### Distribution.

Southwestern China (Yunnan).

##### Remarks.

The diagnostic features of *Mysmenaluosuo* sp. nov. are also largely broad Mysmeninae (Lopardo and Hormiga 2015), but shape of the male palps and the configuration of the vulvae are similar to other species of the same genus (cf. *M.furca* and *M.rostella*), without share features of other genera. Therefore, we propose it as a new species.

#### ﻿ Mysmena rostella

Taxon classificationAnimaliaAraneaeMysmenidae

Lin & Li, 2008

3A9F5D00-6284-5B50-BCA0-29E1C49E377C

Figs 27 , 28 , 29

Mysmena rostella Lin & Li, 2008: 492, fig. 5A–I (♂).

##### Type material.

***Holotype*** ♂ (IZCAS), China: Yunnan, Mengla, XTBG, secondary tropical montane evergreen broad-leaved forest (21.963°N, 101.200°E; 895±10 m), by searching, 6.VIII.2007, G. Zheng leg. Examined.

##### Other material examined.

6♂ 6♀ (IZCAS), China: Yunnan, Mengla, Menglun Nature Reserve, secondary tropical montane evergreen broad-leaved forest (21.913°N, 101.191°E; 880±15 m), by pitfall trapping, 16–31.V.2007, G. Zheng leg.; 2♂ 3♀ (NHMSU), China: same site as for preceding (21.914°N, 101.211°E; 876±15 m), by pitfall trapping, 1–15.IV.2007, G. Zheng leg.

##### Diagnosis.

*Mysmenarostella* is similar to *M.luosuo* sp. nov. in the shape of male palp and the configuration of vulva (cf. Figs 28C, D, 29B, C and Figs 25D, E, 26B, C), but males can be distinguished by having five pairs of modified spines on the chelicerae, three pairs in *M.luosuo* (Fig. 27C vs. Fig. 24C), and by longer embolus extending prolaterally, shorter embolus coils only at the top of bulbus in *M.luosuo* (Fig. 28A, C vs. Fig. 25A, B, D). Females distinguished from *M.luosuo* by the reniform spermathecae, but near globular in *M.luosuo* (Fig. 29C vs. Fig. 26C).

##### Description.

**Male.** See Fig. 27A–D and Lin and Li (2008): 492, 495.

***Palp*** (Fig. 28A–D): orange, comparatively large. Except for retrolateral region, a row of long setae almost encircled the distal brim of tibia (Fig. 28A–D). Cymbium nearly transparent, tip specialized as cymbial conductor, a distal keel on outer wall of cymbium conductor, the cymbial process tip shape, parallel to the cymbial conductor (Fig. 28A–D). Paracymbium big, with long setae (Fig. 28B). Bulb ball shape, embedded in a translucent membranous tegulum. Tegulum with apical apophysis. Embolus long and winding, the tip interacts with cymbial conductor. Spermatic ducts can be seen through tegulum (Fig. 28D).

##### New morphological data.

**Female. *Measurements***: total length 0.57 Prosoma 0.18 long, 0.21 wide, 0.18 high. Abdomen 0.39 long, 0.38 wide, 0.36 high. Clypeus 0.05 high. Sternum 0.16 long, 0.14 wide. Length of legs: I 0.61 (0.17, 0.07, 0.15, 0.12, 0.10); II 0.52 (0.18, 0.08, 0.12, 0.08, 0.06); III 0.52 (0.16, 0.08, 0.12, 0.07, 0.09); IV 0.50 (0.14, 0.08, 0.12, 0.10 0.06).

***Somatic characters*** (Fig. 27E–G). ***Coloration***: prosoma brown-yellow, endites brown, labium white, sternum brown with four yellow spots, ocular base black. Abdomen yellow dorsally, brown ventrally, with white and yellow spots. Legs brown-yellow. ***Prosoma***: carapace nearly pear-shaped. The eight eyes in two rows, AER and PER straight in dorsal view. Chelicerae, endites and labium rectangle, and sternum scutiform, covered with short setae. ***Legs***: number of setae and bristles same as in male, a sclerotized subdistal-ventral femoral spot present at surface of leg I and II. ***Abdomen***: as in male.

***Epigyne*** (Fig. 29A–C): the scape short and thick, and the surface with fine folds (Fig. 29B). Spermathecae small, nearly semicircle. Fertilization ducts short, derived from lateral of spermathecae, twisted anteriorly and then extended to the anterior of spermathecae. Copulatory ducts slightly sclerotized, coiled around the spermathecae, hooklike symmetrically, connected to the ventral of spermathecae (Fig. 29B, C).

##### Distribution.

Southwestern China (Yunnan).

##### Remarks.

The female description of *M.rostella* is provided for the first time.

#### ﻿ Mysmena dai

Taxon classificationAnimaliaAraneaeMysmenidae

Lin & Li sp. nov.

AB19A490-A0C9-5565-9395-38B3F2E4B908

https://zoobank.org/7897FEFB-88B4-418D-BBA0-850F18F33A1F

Fig. 30

##### Type material.

***Holotype*** ♀ (IZCAS), China: Yunnan, Mengla, Menglun, XTBG, primary tropical seasonal rainforest (21.917°N, 101.275°E; 588±17 m), by searching, 19–25.X.2006, G. Zheng leg.

##### Etymology.

The new species is named after the Dai people, an ethnic minority living in Xishaungbanna of Yunnan Province; noun in apposition.

##### Diagnosis.

**Figure 30.**
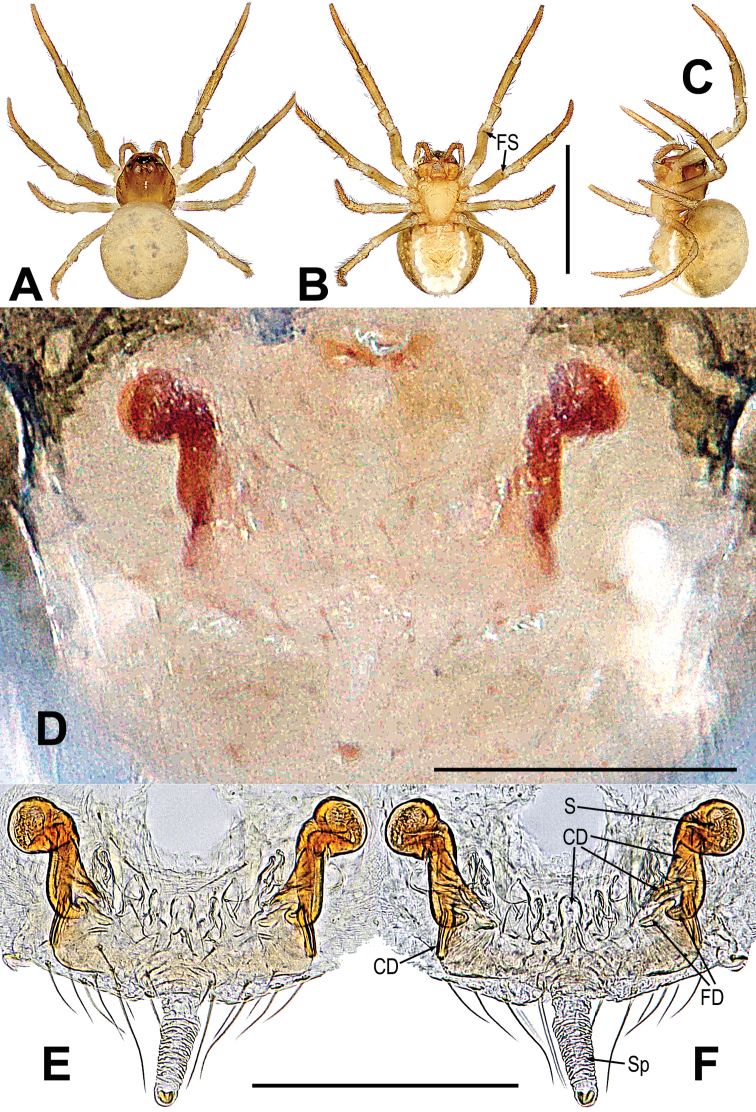
*Mysmenadai* sp. nov. **A–C** female habitus **D** epigyne **E, F** vulva **A, F** dorsal **B, D, E** ventral **C** lateral. Abbreviations: CD = copulatory duct; FD = fertilization duct; S = spermatheca; Sp = scape. Scale bars: 0.50 mm (**A–C**); 0.20 mm (**D–F**).

Females of this new species seems most similar to *M.leucoplagiata* (Simon, 1880) and *M.mooatae* (Baert, 1988) in the configuration of vulva and the rugose long scape, but can be distinguished by the globular spermathecae and the distal end of the descending fertilization ducts, while twisted, ovoid spermathecae, and ascending fertilization ducts in *M.leucoplagiata* (Fig. 30F vs. fig. 11 in Kraus, 1967), semicircle spermathecae in *M.mooatae* (Fig. 30F vs. fig. 24 in Baert, 1988).

##### Description.

**Female** (holotype). ***Measurements***: total length 0.56 Prosoma 0.18 long, 0.23 wide, 0.20 high. Abdomen 0.38 long, 0.30 wide, 0.36 high. Clypeus 0.05 high. Sternum 0.17 long, 0.13 wide. Length of legs: I 0.64 (0.16, 0.08, 0.16, 0.12, 0.12); II 0.51 (0.12, 0.10, 0.15, 0.08, 0.06); III 0.35 (0.10, 0.05, 0.10, 0.06, 0.04); IV 0.47 (0.20, 0.05, 0.10, 0.08 0.04).

***Somatic characters*** (Fig. 30A–C). ***Coloration***: prosoma brown-yellow dorsally, yellow ventrally, ocular base of AER black. Abdomen silver yellow dorsally, yellow ventrally, with “U-shaped” white stripes. Legs brown-yellow. ***Prosoma***: carapace nearly pear-shaped. Eight eyes in two rows, AER and PER straight in dorsal view. Chelicerae, endites as in male, labium triangle, and sternum in the shape of a scutiform, covered with short setae. ***Legs***: covered with setae and bristles. A sclerotized subdistal-ventral femoral spot present at surface of leg I and II. ***Abdomen***: near oval in dorsum, covered with pale short setae.

***Epigyne*** (Fig. 30D–F): the posterior brim with sparse short setae, internal structures visible via translucent cuticle (Fig. 30D). Scape long, with narrow folds (Fig. 30E, F). Spermathecae small, nearly globose, separated by 4× diameter. Fertilization ducts short, derived from lateral of spermathecae vertical posteriorly, curved to the middle distally. Copulatory ducts membranous, connected to lateral margin of spermathecae, fused at the midline position of lower edge of vulva (Fig. 30E, F).

**Male.** Unknown.

##### Distribution.

Southwestern China (Yunnan).

##### Remarks.

The vulva configuration of this species similar to type species of this genus (*M.leucoplagiata* (Simon, 1880)): the presence of scape, the same deriving of fertilization ducts, and same trajectory and extension of copulatory ducts. Therefore, we propose it as a new species.

## ﻿Conclusions

The study on spiders in XTBG were mainly on the following representative families: Araneidae (ex. Mi and Li 2021a, 2021b), Clubionidae (ex. Yu and Li 2019a, 2019b; Zhang et al. 2021a, 2021b), Linyphiidae (ex. Zhao and Li 2014), Pholcidae (ex. Yao et al. 2018; Yao and Li 2018), Theridiidae (ex. Gao and Li 2014), Thomisidae (ex. Tang and Li 2010), and Salticidae (ex. Cao et al. 2016). The investigation about small-size, cryptic symphytognathoid spiders is obviously inadequate. So far, only two anpid species (Lin and Li 2012; Zhang and Lin 2018), five symphytognathid species (Lin et al. 2013; Li et al. 2020, 2021b), four theridiosomid species (Song and Zhu 1994; Zhao and Li 2012) and eight mysmenid species (Lin and Li 2008) were reported.

The current paper draws a general situation of the species composition of the family Mysmenidae in XTBG, and expands the cognition of its mysmenid species diversity. The decision of these new taxa in this study was based on morphological evidences. The next stage of our research will be to verify them by phylogenetic analysis based on molecular evidences.

## Supplementary Material

XML Treatment for Gaoligonga taeniata

XML Treatment for Mengmena

XML Treatment for Mengmena banna

XML Treatment for Mengmena yulin

XML Treatment for Microdipoena menglunensis

XML Treatment for Mosu heguomu

XML Treatment for Mosu zhengi

XML Treatment for Mysmena arcilonga

XML Treatment for Mysmena biangulata

XML Treatment for Mysmena cornigera

XML Treatment for Mysmena furca

XML Treatment for Mysmena luosuo

XML Treatment for Mysmena rostella

XML Treatment for Mysmena dai
